# Data on the stated willingness to accept collective agri-environmental schemes for biodiversity conservation of European grassland farmers

**DOI:** 10.1016/j.dib.2026.112980

**Published:** 2026-06-17

**Authors:** Viviane Fahrni, Robert Finger, Katrin Karner, Martin Schönhart, Sandra Uthes, Claudia Bethwell, Ruth Sophie Hillesund, Klaus Mittenzwei, Gesche Kindermann, Raja Hussain, Stephen Feeney, Michael Joseph Gormally, Caitríona Carlin, Monika Suškevičs, Kalev Sepp, Maaria Semm, Janar Raet, Robert Huber

**Affiliations:** a Agricultural Economics and Policy Group (AECP), ETH Zürich, Sonneggstrasse 33 8092, Zürich, Switzerland; b BOKU University, Department of Economics and Social Sciences, Institute of Sustainable Economic Development, Feistmantelstraße 4 1180, Vienna, Austria; c Federal Institute of Agricultural Economics, Rural and Mountain Research, Dietrichgasse 27 1030, Vienna, Austria; d Leibniz Centre for Agricultural Landscape Research (ZALF), Eberswalder Strasse 84 15374, Müncheberg, Germany; e Geography Department, Humboldt-Universität zu Berlin, Unter den Linden 6 10099, Berlin, Germany; f Ruralis - Institute for Rural and Regional Research, University centre Dragvoll 7049, Trondheim, Norway; g Applied Ecology Unit, School of Natural Sciences, University of Galway, Ireland; h Institute of Zoology, Department of Ecosystem Management, Climate and Biodiversity, BOKU University Vienna, Austria; i Estonian University of Life Sciences, Institute of Agricultural and Environmental Sciences, Chair of Environmental Protection and Landscape Management, Kreutzwaldi 5, Tartu 51006, Estonia

**Keywords:** Choice experiment, Policy preferences, Landscape scale management, Behavioural factors, Collaboration, Cooperation

## Abstract

We present a data set on the stated willingness to accept collective agri-environmental schemes for biodiversity conservation of farmers managing grassland in six European case studies: Austria (AT), Switzerland (CH), Germany (DE), Estonia (EE), Ireland (IE), and Norway (NO). The survey was conducted between spring 2024 and 2025 and consisted of two parts: 1) a choice experiment; and 2) questions about farmers’ and farm characteristics, attitudes, and preferences. In the choice experiment (Part 1), farmers were presented with 10 consecutive choice tasks. Each task consisted of three alternatives, two hypothetical designs for collective biodiversity conservation schemes at the landscape scale and an opt-out option, representing the status quo. The method helps to clarify farmers’ policy design preferences and willingness to accept (WTA) collective biodiversity conservation schemes. Non-monetary attributes of the choice experiment included 1) group size (i.e., the number of partners a farmer works in a collective scheme with), 2) monitoring (i.e., execution of the biodiversity monitoring process), and 3) discretion (i.e., responsibility for management decisions at the farm to enhance biodiversity conservation: the farmers or an external consultant). The survey questions (Part 2) collected data on farmers’ personal and farm characteristics, production orientation, attitudes towards collaboration, the environment, trust and self-efficacy, their risk, time, and social network preferences, as well as their income satisfaction. To reduce respondents' load, the survey data was matched with farm census data in instances where such data was available (CH, AT, NO).

Specifications Table

**Table:** 

Subject	Social Sciences
Specific subject area	We present data on farmers’ stated policy design preferences for collective agri-environmental schemes combined with socio-economic and behavioural factors.
Type of data	Table, Filtered, Processed, Anonymized
Data collection	Surveys were collected via the online platform LimeSurvey (www.limesurvey.org). The choice experiment was designed in accordance with current literature to answer the research questions. Question items were determined through literature review and follow previous examples.
Data source location	Data was collected in six case study regions: • Austria, all of Austria with a focus on grassland farms • Switzerland, Canton of Grisons • Germany, all of Germany, only farms with grassland • Estonia, West-Estonia (Lääne, Pärnu, Saare, Hiiu Counties, and western municipalities of Harju County) • Ireland, Agri-Climate Rural Environment Scheme (ACRES), Corncrake Life project study and Lough Carra Life project farms • Norway, all grassland farms in Norway with a focus on Valdres The data is stored in [1].
Data accessibility	Repository name: ETH Research Collection Data identification number: https://doi.org/10.3929/ethz-c-000795442 Direct URL to data: https://www.research-collection.ethz.ch/entities/researchdata/5312febb-7af2–4709-a06d-efe2b53574f1 Instructions for accessing these data: The six data sets are linked together in the data collection specified by DOI and URL above. The data is publicly available at the ETH Research Collection.

## 1 Value of the Data

• The data can be used to elicit farmers’ stated preferences for different characteristics of collective biodiversity conservation schemes. The data provides the basis for the analysis of behavioural factors that influence farmers’ uptake of collective biodiversity conservation schemes.
• The dataset will be important for researchers and policy makers who want to understand the factors driving farmers’ willingness to participate in collective biodiversity conservation schemes.
• Data allows the comparison and analysis of behavioural factors in the adoption decision of collective agri-environmental schemes as well as analyses of farmers’ willingness to accept collective agri-environmental schemes across European case study regions and countries.
• Data can be used in meta-analysis of farmers’ behaviour and the assessment of theoretical models of farmer behaviour.
• Data can support the design of new and optimised collective agri-environmental schemes.

## 2 Background

Ecosystems are deteriorating and biodiversity is lost at increasing rates worldwide and agricultural production is a key driver of this development. Agri-environmental schemes are a major policy instrument to incentivize farmers to adopt practices that support biodiversity conservation. However, agri-environmental schemes are not currently considered to be effective in meeting European policy goals. At the same time, landscape-level management including collective work of farmers in collective agri-environmental schemes is seen as a promising way to ensure more effective and efficient biodiversity conservation. However, the success of landscape-level management in agri-environmental schemes often depends on the specific context and the design of the scheme and we currently lack understanding of how socio-economic and behavioral factors influence the adoption of collective agri-environmental schemes across different farming contexts and countries. Thus, the data was collected to analyze farmers’ willingness to accept collective agri-environmental schemes in combination with socio-economic and behavioral factors across six European case studies.

## 3 Data Description

The survey data can be found in a data collection [1]. The data collection links the six case study specific data sets. To clarify the case study specific differences and contexts codebook files give detailed information about every variable and the formulation of questions used to obtain the data. PDF files of the surveys are available in the respective original languages as well as a version translated to English per case study.

## 4 Experimental Design, Materials and Methods

### 4.1 Data collection

The survey was conducted using the online platform LimeSurvey (www.limesurvey.org). The questionnaire was based on literature review and discussions within the project consortium.

The survey was pre-tested in all case study regions to ensure understanding of the survey and validate the choice experiment attribute levels. In Austria, 24 students from an agricultural high-school and their farming parents pre-tested an initial version in January 2024. In Switzerland pre-testing was done with 17 farmers in master craftsmen training at the agricultural school Strickhof Wülflingen 08.12.2023. The pretesting in Germany is described below together with the data collection procedure. In Estonia, the pre-testing was done on the 6th and 16th of May 2024 with two representatives from the Ministry of Regional Affairs and Agriculture. Pretesting in Ireland was done with five people of the Corncrake project team at the University of Galway on the 22nd of April 2024. In Norway pre-testing was done with 7 students at the agricultural school Skjetlein 14.12.2023. The pre-testing results from Switzerland were furthermore used as basis for the calculation of the final efficient choice card set (See Table 1) using the modified Fedorov algorithm [2]. According to the power calculations [3,4] based on the Swiss pre-testing results a minimum of 100 responses were required per case study region for meaningful quantitative econometric analysis (aspired *n* = 150 farmers per case study region) (See Table 2).

**Table 1:** d-efficient choice experiment design.

	Opt-Out	Payment	Group Size	Monitoring	Discretion
set1.alt1	0	500	20	1	0
set1.alt2	0	500	20	1	1
no.choice.1	1	0	0	0	0
set2.alt1	0	1000	1	0	0
set2.alt2	0	1000	1	1	1
no.choice.2	1	0	0	0	0
set3.alt1	0	500	6	1	0
set3.alt2	0	1000	20	1	0
no.choice.3	1	0	0	0	0
set4.alt1	0	1000	20	1	0
set4.alt2	0	500	10	0	0
no.choice.4	1	0	0	0	0
set5.alt1	0	1000	3	1	1
set5.alt2	0	1000	1	1	1
no.choice.5	1	0	0	0	0
set6.alt1	0	500	6	0	0
set6.alt2	0	500	3	1	1
no.choice.6	1	0	0	0	0
set7.alt1	0	500	1	1	1
set7.alt2	0	1500	10	1	1
no.choice.7	1	0	0	0	0
set8.alt1	0	1000	20	0	0
set8.alt2	0	500	1	1	0
no.choice.8	1	0	0	0	0
set9.alt1	0	500	1	0	1
set9.alt2	0	500	3	1	0
no.choice.9	1	0	0	0	0
set10.alt1	0	1000	20	1	0
set10.alt2	0	1000	1	0	1
no.choice.10	1	0	0	0	0

		Continuous	Continuous	1 = In-person	1 = State
				0 = Digital tools	0 = Farmers

**Table 2:** Power calculations.

	Opt-Out	Payment	Group Size	Monitoring	Discretion
Priors (rounded)	−1.941	0.001	−0.338	0.281	−0.688
Required N	7	49	2	99	22

The timeframe and scope of data collection in the case study regions was as follows:

In Austria, a random sample of grassland farmers received an Email-invitation for the online survey. The Federal Institute of Agricultural Economics, Rural and Mountain Research randomly sampled 3000 farmers who had at least 50% grassland in their total agricultural area across Austria. The survey was online from 8th of March 2024 to 30th of June 2024.

On the 12th of February 2024 an invitation to the online survey was sent to 2587 e-mail addresses of farmers of the Canton of Grisons, Switzerland, (all farmers of whom the e-mail address was available through census data, including managers of year-round farms, summering farms, and farming cooperations.). Two reminders were sent. The survey was online until the 8th of April 2024.

In Germany, the farm structure of the focus region Havelland with comparatively few farms was unlikely to meet the minimum required number of respondents. Thus, the online survey was carried out by a marketing research company that also did the pre-testing directed at grassland farmers throughout Germany. The farm survey was sent to 10.000 farms managing grassland on the 10th of July 2024, and the survey was closed when the required response rate was achieved, 22.07.2024.

In Estonia, initially, an email invitation for the online survey was sent out to 1525 agricultural support applicants in Lääne County and Pärnu County on May 16, 2024. The second reminder was sent on June 27, 2024, and the third on September 20, 2024, only to participants who have started, but not fully completed a survey. Due to the low response rate, we sent the survey to an additional 1424 agricultural support applicants in Hiiu County, Saare County, and the western municipalities of Harju County on November 18, 2024. A reminder for this group was sent on December 18, 2024. Both groups included applicants who applied for grassland subsidies of over 1 hectare in 2023. The farmers’ contact data used is managed by the Agricultural Registers and Information Board (ARIB). The response rate for the first group was 64, while for the second group it was 42. A final reminder was sent to the farmers who had started the filling-in but had not completed it on January 27, 2025. The survey was closed February 20, 2025.

In Ireland the survey was presented in-person to the farmers at knowledge exchange events organized by the Corncrake LIFE project. As sufficient numbers were not met by the Corncrake farmers alone, the study region for the questionnaire was expanded from the focus region to the rest of Connacht, the western province in Ireland. A Lough Carra LIFE project knowledge exchange event and several Agri-Climate Rural Environment Scheme (ACRES) knowledge exchange events were attended by GreeNet members where the questionnaire was again presented to the farmers in the same manner. Survey data was thus collected between 03.09.2024 and 14.12.2024.

In Norway, the online survey was sent out early February to 3000 farmers including all farmers with grassland in Valdres and randomly selected farmers outside the focus region. A reminder was sent out one time. Responses were collected between 14.02.2024 and 14.03.2024.

The case study regions obtained the following number of full responses (the survey was completed) respectively: Austria 195; Switzerland 206; Germany 173; Estonia 106; Ireland 132; Norway 141 (Total *N* = 953). To ensure the validity of the responses we asked whether the choice experiment was understood by the participant, and only considered respondents who did not systematically opt out. Removing invalid answers leads to final sample sizes as follows: Austria 135; Switzerland 123; Germany 143; Estonia 64; Ireland 104; Norway 67 (Total *N* = 636).

### 4.2 Data processing

The raw data from LimeSurvey was filtered, processed, and anonymized in the following ways:

- LimeSurvey specific variables that are not relevant to the collected data were removed (timestamps, tokens, e-mail addresses, empty fields from text boxes).
- Column and variable names/codes were harmonized across different case study regions.
- Incomplete responses were removed. Responses were considered as complete, if all questions including those about production orientation were answered. Free text comments and miscellaneous questions in the outro did not need to be filled in for the response to count as complete.
- Several sensitive answers were coded as 1 (numeric) or Y (character) for all respondents for reasons of data privacy. Open text questions and ZIP codes were considered as sensitive.

### 4.3 Case study region descriptions

**Fig. 1:**
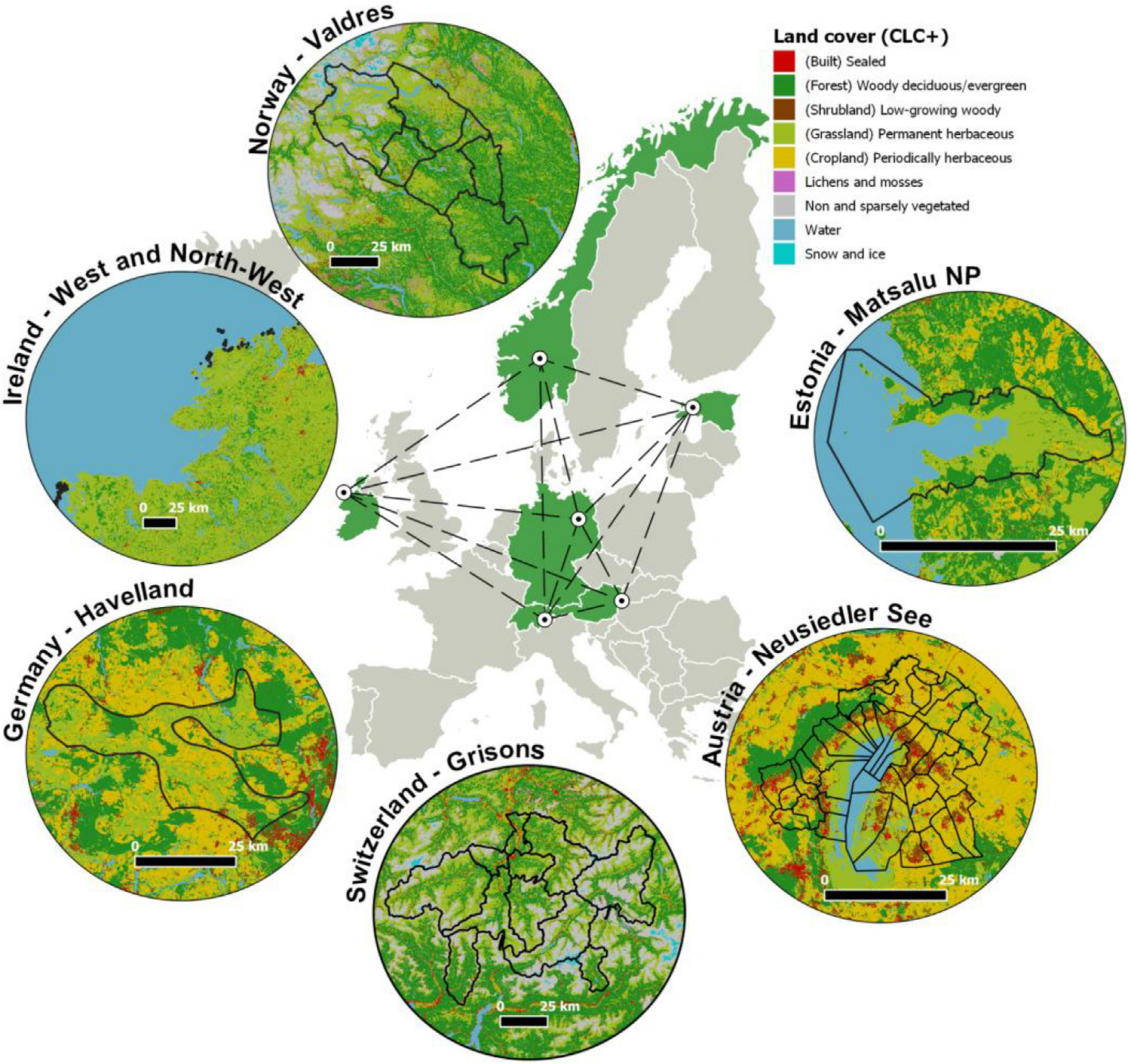
GreeNet partners and their respective focus regions. Anti-clockwise: Valdres Norway (NO), West and North-West Ireland (IE), Havelland Germany (DE), Grisons Switzerland (CH), Neusiedler See Austria (AT), and Matsalu NP Estonia (EE).

We present data on the stated willingness to accept (WTA) collective agri-environmental schemes for biodiversity conservation from farmers in six grassland-dominated case study regions: Austria, Switzerland, Germany, Estonia, Ireland, and Norway (see Fig. 1). The six case study regions were chosen based on the research project’s consortium structure. They correspond to the participating countries in the research project. In all six countries a focus region was chosen for the research project. To collect the survey data presented in this article, focus regions were partially extended to ensure a large enough sample size. Extension was done in one of two ways: 1) The focus region plus surrounding regions were chosen as the case study region (Estonia, Ireland, Norway), or 2) a national roll-out was done (Austria, Germany). The resulting case study regions are composed as follows:

For Austria the region around Neusiedler See was chosen as the focus region. For the survey a random sample of grassland farmers across Austria who had at least 50% grassland of their total utilised agricultural area were contacted. Austria is characterised by a rather small structured agricultural sector with a large variety of production regions - ranging from very productive cropland to alpine grassland areas. In total, 110.781 farms manage 1.32 million ha of arable land, of which 216,983 ha are field forage production, and 1.21 million ha of permanent grassland (excluding alpine meadows). 3.41 million ha are forests. A total of 26% of farms are livestock farms, with, e.g., an average of 34 cattle or 23 dairy cows. 44% of farms are part-time farms. The mean farm size is 23.6 ha (agricultural utilized area). 23% of all farms are organic farms, which in total manage 27.4% of the agricultural utilized area in Austria. In total, 84% of farms participate in one measure offered in the Austrian Agri-environmental programme ÖPUL.

For Switzerland the canton of Grisons was chosen as the focus region and case study region for the survey. It includes the Swiss National Park (nature reserve), the UNESCO biosphere Val Mustair, and the Swiss parks Beverin and Ela. The canton to the south-east of Switzerland is characterized by alpine landscapes, with more extensive meadows, summering pastures, and forests on the mountain slopes, and more intensive grassland use in the valleys. 95% of the agricultural land use in the canton is committed to grassland production, with only a low percentage of area used for arable farming or permanent crops. Grisons is unique in the Swiss agricultural context due to a high share of organic farms (59%) and high share of organically farmed land (65.8%). The mean farm size is 26 ha (32 ha for farms where farming is the main occupation).

The German focus region was the natural area of Havelland, which is located in the northeast of Germany near Berlin. For Germany, a national roll-out was done as the initially selected focus area of Havelland (‘Luchland’) had comparatively small farm numbers, so that it was perceived unlikely that the sample size required for the choice experiment could be met. The land use types in Germany are approximately 50% agricultural land, 30% forests, 15% settlement and traffic areas, and 5% other vegetation areas. Approximately 29% of the agricultural area in Germany is permanent grassland, i.e.4.7 million hectares managed by 219,883 farms. The mean farm size in Germany is 63 ha, whereby the farms range from <5 ha to >200 ha; most farms are in the range of 20 to 50 hectares (23% of farms) and the largest utilised agricultural area is in the range of 200 hectares and above (41% of the area). Organic farming is conducted in 9.9% of the farms and on 9.6% of the utilised agricultural area. Protected areas in Germany are nature protection areas, landscape protection areas, biosphere reserves, nature parks and Natura 2000 sites.

The Estonian case study region for the survey, West Estonia, includes the focus region Lääne County and extends to West Estonia in a larger sense, i.e. Pärnu County and western municipalities of Harju County (Lääne-Harju, Saue, Harku, and Saku municipalities), plus Hiiu and Saare Counties. With its large share of semi-natural grasslands and belonging to the most species-rich areas of Estonia, West Estonia is suitable as a case study region. Forest is the predominant land use type in all counties. The share of grassland is approx. 6.9–17.1%. The case study region includes protected areas such as national parks, nature reserves, landscape protection areas and Natura 2000 sites. Farm sizes range from 0,5 to 5000 hectares, with a mean of 217 hectares. 14% to 70% of the area is managed organically in the different counties.

The Corncrake LIFE project area was chosen as the Irish focus region. This project focuses on a network of low-lying, coastal farmland, stretching across the West and North-west of Ireland, in counties Galway, Mayo and further north to Donegal. Farmers from these regions are incentivized with results-based payments by the Corncrake LIFE project to remain committed to low-intensity, traditional methods of farming. The mean contracted parcel size is 1.15 ha. Roughly 95% of the farmland is used for livestock, either directly for grazing or indirectly for silage/hay production. Around 16% of the Corncrake LIFE farmers are in organic production, with about 20.7% of land being farmed organically. Additionally, farmers from two other agri-environmental schemes were interviewed, ACRES and Lough Carra LIFE. The regions in which these schemes operate are comparable landscapes to the Corncrake LIFE project areas but are situated slightly further south. ACRES is Ireland’s largest agri-environmental climate initiative which again aims to combat biodiversity declines by funding >50,000 farmer participants. In the same vein as the Corncrake LIFE project, results-based payments are used to incentivize these farmers to follow a sustainable plan for their farm, approved by an agricultural Advisor. Lough Carra LIFE is another incentivised agri-environmental scheme aimed at improving water quality and restoring habitat in the Lough Carra catchment area. The project also collaborates with farmers, landowners and local community groups.

The Valdres region was chosen as the Norwegian focus region. This large valley covers the municipalities Sør-Aurdal, Nord-Aurdal, Etnedal, Vestre Slidre, Øystre Slidre and Vang. In the bottom of the valley the landscape is dominated by agriculture, with the surrounding hills covered by boreal forest, while the northern part is mainly alpine. Traditional summer-farming practices are still maintained in parts of the sub-alpine areas. Calcareous bedrock in parts of the region supports a rich plant and insect diversity. Almost all agricultural area is grassland, and the small area for arable farming has even declined during the past ten years. The area borders the Jotunheimen National Park but has no protected areas itself. The average farm size is about 20 ha. About 4 per cent of the agricultural area qualifies for organic production. The region is typical for Norway where grasslands and ruminant production is characterized by intensive production in the valleys and extensive grazing in the mountains.

### 4.4 Census data

To reduce respondents' load, the survey data was matched with farm census data in instances where such data was available (CH, AT, NO). This concerned the following variables (see Table 3):

**Table 3:** Census data use in case study regions.

Variable	Austria (AT)	Switzerland (CH)	Norway (NO)
FarmSize	x	x	x
ZIP	x	x	x
WorkForce		x	
PaymentSchemes	x	x	
PaymentArea	x	x	

In Austria the census data was sourced from the national Integrated Administration and Control System (IACS), which is used to administrate the EU Common Agricultural Policy in Austria. In Switzerland the census data was sourced from the Cantonal office of agriculture and geoinformation of the Canton of Grisons upon request. In Norway, the census data with animal and crop activity data for every farm eligible for payments was sourced from the public agency Data Norway. The data contains the farm’s georeferenced at the municipality level and is accessible without cost.

Using census data in some case study regions but not in others reduces comparability between the case study regions. On one hand, census data likely is more accurate than self-reported values from a survey and while it may decrease measurement error within a case study region this is not the case between case studies. Since likely there are differences between the countries anyways, we do not expect a systematic bias. On the other hand, respondents who need to answer more questions may lose more focus by the end of the survey compared to respondents who answer fewer questions. The result would be an increase in measurement error in countries with longer surveys where census data is missing. However, this risk was deemed acceptable and given the length of the survey, reducing the respondents’ load was a priority.

### 4.5 Choice experiment

The core of the survey consisted of a choice experiment with 10 choice cards. The non-monetary attributes of the choice experiment were determined through a literature review, and included: 1) group size (i.e., the number of partners a farmer works in a collective with [5]), 2) monitoring process, and 3) discretion (i.e., who is responsible for the design of the management decisions to enhance biodiversity conservation (see Table 4). Examples of the monitoring options can be found in Elmiger et al. [6]. The attribute discretion is based on the paper by Prager [7]. All three attributes reflect farmers’ expected transaction costs under a collective biodiversity conservation scheme [8]. An example choice card is shown in Table 5.

**Table 4:** Choice experiment attributes and attribute levels.

Attributes	Levels	Nr. levels
Additional Payments	All: Low, medium, high AT: 700, 1000, 1300 Euro/ha/year CH: 500, 1000, 1500 CHF/ha/year DE: 500, 1000, 1500 Euro/ha/year EE: 300, 600, 1000 Euro/ha/year IE: 30, 60, 90 Euro/ha/year NO: 500, 1000, 1500 NOK/ha/year	3
In group with	1, 3, 6, 10, 20	5
Monitoring	Expert in-person, Data collection via digital tools	2
Discretion	(Local) Government, Farmer’s collective	2

**Table 5:** Example Choice Card, here for the Swiss case study region.

		Option A	Option B	None of the two
	**Additional Payments** compared to current levels	+1000 CHF per ha and year	+500 CHF per ha and year	No change
	**Number** of farmers you are in a group with	20	10	No change
	**Monitoring** in person or via digital tools	In person	Digital tools	No change
	**Discretion** with the local government or farmers collective	Farmers	Farmers	No change
**Your Choice**		□	□	□

The ten choice cards of the choice experiment were followed by a set of debriefing questions [9]. Respondents were asked whether the explanation of how the choice experiment works, what the collective scheme would entail and what the choice options are, was clear to them. If a respondent for all 10 choice cards systematically chose the opt out (no change) option, they were asked for their reasoning: a) the payment being too low, b) a goal mismatch between the scheme and their own goals, c) the scheme being unrealistic, d) that they refuse any inference with their management, e) no reason, f) other reasons. All participants were asked whether they always, sometimes, or never ignored any of the four attributes, to account for attribute nonattendance [10]. Assuming the “best” circumstances (“best” in the sense that if the scheme were to be implemented according to the preferences they had just revealed), respondents were asked on how much of their farm area they would be willing to implement such a scheme [11]. Additionally, participants were asked what they considered to be likely outcomes (perceived costs and benefits) of such a collective agri-environmental scheme under the “best” circumstances on a five-point scale from “does not apply at all” to “ applies very much”: a) additional payment would compensate the additional effort, b) additional payment could be obtained without additional effort, c) production would increase, d) production would decrease, e) there would be collective learning and exchanges with other farmers, f) personal agency and control over own farm management would be lost to the group, g) one could show one's skills as a farmer, h) biodiversity levels would increase, i) production systems would become more resilient, j) as a group, farmers could diversify or mobilize other resources, k) as a group farmers would have more weight in negotiations, l) one would spend much time and energy on the scheme, or m) other implications [7].

### 4.6 Farmer and farm characteristics

In the questionnaire farmers were asked about their personal and farm characteristics, including gender, age range, year of taking over or starting the farm, education level, and farm succession, adapted from [[12], [13], [14]]. We elicited the following farm characteristics: farm size (total area in ha), postal code,^1^ labour unit, percentage of leased land, production form, participation in voluntary payment schemes relating to biodiversity conservation or collective management and area covered under these schemes, farmland in protected areas and the type of protection status, adapted from [14,15]. The questions regarding voluntary payment schemes and types of protected areas were adapted to fit the context of the different case study regions.

### 4.7 Attitudes towards cooperation

As part of the questionnaire, farmers were asked about their attitudes towards cooperation and their cooperation experiences and intentions. On a five-point scale from “strongly disagree” to “strongly agree”, participants evaluated the following statements: 1) I enjoy working with other farmers, 2) Achieving environmental goals in agriculture requires greater cooperation among farmers, 3) Cooperation with other farmers is generally difficult, and 4) I frequently exchange ideas with other farmers on agricultural topics [16]. Survey respondents were asked to indicate their cooperation experience and intentions a) in the past, b) ongoing, c) planned or considered for the future, and d) never / not considered, across different domains: 1) environmental management, 2) machinery sharing, 3) summering, 4) input purchases, sales, and processing, 5) shared field work, 6) looking after someone else’s farm, 7) exchanging knowledge and shared learning, 8) alps (only AT), 9) pastures (only AT), and 10) other domains.

### 4.8 Attitudes towards the environment

Farmers were asked about their attitudes towards the environment with a focus on biodiversity. On a five-point scale from “strongly disagree” to “strongly agree”, they were asked to indicate how much they agree with statements on global, regional, and farm scale respectively regarding biodiversity declining, biodiversity decline being an issue, and biodiversity being important to them [14,15]. Additionally, survey participants were asked if there is a specific aspect of biodiversity that is important to them, and if yes, which.

### 4.9 Self-efficacy, locus of control

To elicit a measure for locus of control and self-efficacy, respondents were asked to evaluate the following statements on a five-point scale from “strongly disagree” to “strongly agree”: 1) I can do something about biodiversity decline on my farm, 2) When I encounter difficulties in my production, I can usually think of a solution, 3) My behaviour as a farmer influences biodiversity, 4) How successfully I can reduce biodiversity decline on the farm depends mainly on my skills as a farmer, 5) I can solve production issues if I invest the necessary effort, 6) I am confident that I can reduce biodiversity decline and at the same time produce successfully, and 7) Biodiversity decline is a problem I cannot change, adapted from [12].

### 4.10 Risk and time preferences

We elicited risk and time preferences with an eleven-point Likert scale going from “not willing” to “very willing”. Participants were asked to evaluate their willingness to take risks in the four domains production, market and prices, agriculture in general, and collaboration [17,18]. Survey respondents indicated their time preference by stating their willingness to give up income that would be beneficial to them or their farm today in order to benefit more from that in the future [17].

### 4.11 Trust

Participating farmers were asked to indicate their trust towards 1) people in general, 2) farmers in their region, and 3) the regional government, by evaluating whether these groups only have the best intentions on a five-point scale from “strongly disagree” to “strongly agree” [15,17].

### 4.12 Social network preferences

We elicited farmers’ social orientation, social network, and social network importance. For social orientation survey respondents were asked to evaluate the following statements on a five-point scale from “strongly disagree” to “strongly agree”: 1) It is important to me what people around me think about the success of my farm and my farming skills, 2) it is important to me to impress other farmers with my farm, 3) I feel confirmed if I earn more than other farmers, 4) on my farm, I want to produce more environmentally friendly than other farmers in my area, 5) if other farmers in my environment earn more than I do, it bothers me, and 6) if other farmers in my environment implement biodiversity conservation measures, I want to implement such measures on my farm as well [12]. To understand farmers’ social network we asked with how many people in the following groups respondents had discussed agricultural topics and biodiversity conservation measures within the last year: a) Other Farmers / people working in agriculture / association colleagues, b) Consultants/Extension service, c) Biodiversity Experts / Representatives of environmental organizations, d) Policy makers, e) Non-farmers in my community, and f) Customers (direct marketing or retailers), adapted from [12]. To elicit the importance of these social connections we asked farmers to evaluate how important the opinions of these groups of people are to them on a five-point scale from “not at all important” to “very important”, adapted from [12].

### 4.13 Income satisfaction, income importance, and production orientation

We elicited farmers’ income satisfaction, income importance and production orientation. For income satisfaction survey respondents were asked how satisfied they are with their farm income on a five-point scale from “not at all satisfied” to “very satisfied” [12]. The importance of agricultural income was elicited on a five-point scale from “not at all important” to “very important”. The share of agricultural income in total household income was elicited as percentage ranges: 0–20%, 21–40%, 41–60%, 61–80%, and 81–100% [12].

We elicited production orientation by asking responding farmers to evaluate the importance of the following 16 criteria for decision making on a five-point scale from “not important at all” to “very important”: 1) ensuring greatest crop yield, 2) forestalling future problems, 3) professional ambition to use best practices, 4) contribution to a healthy environment, 5) price of fodder, 6) clean fields (few weeds visible), 7) reduction of own workload, 8) costs of inputs, 9) subsidies for implementing sustainable methods, 10) keeping a high biodiversity on fields, 11) achieving a high income (including direct payments), 12) own image as a “good” farmer, 13) consumers’ demand for products produced with a certain standard, 14) long-term increase of soil fertility on own agricultural land, 15) other farmers’ management methods, 16) traditions, adapted from [19].

## Limitations

There are slight differences between the surveys in the six case study regions due to translation from English (which was used during the design of the survey) to the country-specific national languages, specific technical terms, and to reflect the different agricultural, political, economic, and environmental contexts. For instance, while collective agri-environmental schemes have been implemented in Switzerland for quite a while, no such schemes yet exist in Norway. One limitation of the survey data is the staggered roll-out of the survey in the different case study regions (see EXPERIMENTAL DESIGN, MATERIALS AND METHODS, Data collection). Different means of contacting farmers for survey participation could also potentially limit the quality of the data: via E-Mail (CH, AT, NO, EE), via a marketing research company (DE), or in-person in regular meetings (IE).

## Ethics Statement

We hereby confirm that the relevant informed consent was obtained from the test subjects (survey participants, consisting of farmers and farm managers). The survey was pre-registered (AsPredicted, # 161,542) and obtained ethical clearance from the ETHZ Ethics Commission as call EK-2023-N-322 before roll-out. The ethical clearance obtained from the ETHZ Ethics Commission encompasses survey implementation in all case study regions. However, wherever possible clearance from other national ethics commissions was also obtained. In Germany, the Ethics Commission of the Leibniz-Zentrum für Agrarlandschaftsforschung (ZALF) e.V. accepted the survey as issue 2024–06–21. In Norway, the legal basis for the survey was given by the Norwegian Agency for Shared Services in Education and Research under reference number 901,807.

## CRediT Author Statement

**Viviane Fahrni:** Conceptualization, Data curation, Methodology, Formal analysis, Writing - Original draft preparation; **Robert Finger:** Supervision, Writing - Reviewing and Editing; **Katrin Karner:** Data curation, Writing - Reviewing and Editing; **Martin Schönhart:** Project administration (of GreeNet, and of the Austrian team), Funding acquisition (from the Austrian Science Fund), Writing - Reviewing and Editing; **Sandra Uthes:** Data curation, Writing - Reviewing and Editing; **Claudia Bethwell:** Data curation, Writing - Reviewing and Editing; **Ruth Sophie Hillesund:** Data curation; **Klaus Mittenzwei:** Project administration (of the Norwegian team), Funding acquisition (from the Research Council of Norway), Supervision, Writing - Reviewing and Editing; **Gesche Kindermann:** Data curation, Writing - Reviewing and Editing; **Raja Hussain:** Data curation, Writing - Reviewing and Editing; **Stephen Feeney:** Data curation, Writing - Reviewing and Editing; **Michael Joseph Gormally:** Project Administration (of the Irish team), Funding acquisition (from the Environmental Protection Agency), Writing - Reviewing and Editing; **Caitríona Carlin:** Data curation, Writing - Reviewing and Editing; **Monika Suškevičs:** Project administration (of the Estonian team), Data curation, Writing - Reviewing and Editing; **Kalev Sepp:** Project administration (of the Estonian team), Funding acquisition (from the Estonian Research Council), Data curation, Writing - Reviewing and Editing; **Maaria Semm:** Data curation, Writing - Reviewing and Editing; **Janar Raet:** Data curation, Writing - Reviewing and Editing; **Robert Huber:** Project administration (of the Swiss team), Funding acquisition (from the Swiss National Science Foundation), Conceptualization, Methodology, Supervision, Writing - Reviewing and Editing.

## Data Availability

(ETH Research Collection).Datacollection on the stated willingness to accept collective agri-environmental schemes for biodiversity conservation of European grassland farmers (Original data).

## Appendix Supplementary materials

Image, application 1

Image, application 2

Image, application 3

Image, application 4

Image, application 5

Image, application 6

Image, application 7

## Footnotes

1 Exception Ireland: In Ireland county names were used to ensure better recognition and relevance for respondents, as counties are a more commonly used and understood geographical reference. Exception Germany: In Germany the federal state names were used as geographical reference.
