# Supplementary material for: Data on the stated willingness to accept collective agri-environmental schemes for biodiversity conservation of European grassland farmers
Source: Data Brief. 2026 Jun 17;67:112980. doi: 10.1016/j.dib.2026.112980 (PMC13315105; doi:10.1016/j.dib.2026.112980)
Supplement: Supplementary file 1 [file mmc1.pdf]

CONFIDENTIAL - FOR PEER-REVIEW ONLY

GreeNet Farmer Survey (#161542)

Created: 02/09/2024 07:01 AM (PT)

This is an anonymized copy (without author names) of the pre-registration. It was created by the author(s) to use during peer-review.
A non-anonymized version (containing author names) should be made available by the authors when the work it supports is made public.

1) Have any data been collected for this study already?
No, no data have been collected for this study yet.

2) What's the main question being asked or hypothesis being tested in this study?
We intend to do a survey including a choice experiment (CE). The choice experiment elicits farmers' willingness to accept a collective agri-environmental scheme (AES) for biodiversity conservation. The rest of the survey elicits farmer and farm characteristics, farmers' preferences, and attitudes.
The two main hypotheses are:
1)Farmers' willingness to accept a collective AES is lower (i.e., they require higher payment to participate) if transaction costs of the scheme are higher. Transaction costs are represented by three aspects, i.e., i) group size of farmer collectives (attribute "Collective"), ii) planning work done by farmers (attribute "Discretion") and iii) time spent by farmers on monitoring (attribute "Monitoring").
2)Farmers have heterogenous preferences for AES attributes and can be grouped by latent class analysis. This heterogeneity can be explained by social, cognitive, and dispositional factors of the farmers and the social interaction with their peers.

3) Describe the key dependent variable(s) specifying how they will be measured.
The dependent variable is farmers' willingness to accept (WTA) a given collective AES (i.e., given specific combinations of CE attribute levels). WTA will be elicited by repeatedly (10 times) choosing an option on choice cards with different combinations of CE attribute levels.

4) How many and which conditions will participants be assigned to?
Participants are asked to fill out ten choice cards in the CE. Each choice card has three options, i.e., two (A and B) representing combinations of the attribute levels, and an option (C) which represents an opt-out. Efficient choice set design has been calculated following de Bekker-Grob et al. (2015) and Pérez-Troncoso (2020) and power calculations following the same authors resulted in 10 choice cards for a response rate we think we can reach (see point 7).
Apart from that participants are asked to give information about various characteristics, preferences, and attitudes (see point 8).

5) Specify exactly which analyses you will conduct to examine the main question/hypothesis.
The choice experiment will be analyzed using a mixed logit model (following e.g., Zemo and Termansen (2018)) or latent class analysis (following e.g., Kreft et al. (2021)).

6) Describe exactly how outliers will be defined and handled, and your precise rule(s) for excluding observations.
As the survey is sent out to our target-group only, all complete surveys can be considered as observations. Responses will be excluded if participants indicate that they did not understand the choice experiment. Responses will be categorized as protest-votes if participants opt out for all choice cards without giving a reason in the de-briefing questions. Protest-votes will be analyzed but excluded in the main analysis. Additionally, we use a multivariate outlier identification procedure (e.g., Billor et al. 2000; Béguin and Hulliger 2008) to identify outlying observations (dependent and potential control variables).

7) How many observations will be collected or what will determine sample size? No need to justify decision, but be precise about exactly how the number will be determined.
The survey will be held in 6 case study regions by different project partners, namely Norway, Ireland, Estonia, Germany, Austria, and Switzerland. According to power-calculations (see point 4) done after pre-testing in Switzerland, 99 responses per case study region are enough to detect all effect sizes in the CE when 10 choice cards are used. Therefore, all project partners aim for at least 100-150 complete survey responses in their respective case study region. We expect a response rate of 10-15% and therefore target 1000 farmers or more in each case study region.
All project partners contact farmers within the region (for example Val Müstair in Switzerland) and expand to nearby regions (for example the entire Canton of Grisons) if not enough farmers live in the case study region itself. For the Austrian case study farmers across Austria will be contacted to ensure the expected response rate is met.
All project partners contact farmers who manage grasslands; the case study regions were chosen along a gradient of different grasslands and different protection statuses at the outset of the project. We exclude farms that only manage arable crops or livestock and do not manage any grasslands.
Where e-mail addresses are available the survey link will be sent out to farmers via e-mail, for example in Switzerland to all active farmers of the canton of Grisons which have applied for direct payments (i.e., professional farms) or are leading a summering farm (different farm structure but concerned with grasslands) in 2023 (~2500). Alternatively, project partners will contact farmers by other means such as in person at agricultural fairs or via postal services.
Sample size will be the number of complete survey responses we receive, minus exclusions (see point 6). Therefore, sample size is determined by our

conditions for sending out the survey and self-selection of the farmers who chose to answer the survey completely.

8) Anything else you would like to pre-register? (e.g., secondary analyses, variables collected for exploratory purposes, unusual analyses planned?)
In the survey data on preferences towards agricultural practices and some farm information will be collected.

Namely:
• Personal characteristics (age, gender, education, farm succession)
• Farm characteristics
• Preferences in the choice experiment
• Attitudes towards cooperation
• Attitudes towards the environment
• Attitudes towards self-efficacy
• Risk and time preferences
• Attitudes towards trust
• Social network preferences
• Income satisfaction and production orientation
• Comments

Where available the survey responses will be matched with census data, e.g., in Switzerland census data was provided by the Canton of Grisons for the purpose of this study. Data use contracts for using this data have been drawn up. This data set includes spatially explicit farm information on participation in biodiversity conservation schemes as well as farm structural data, and personal data (phone number, address, and e-mail address). Access to census data enables project partners to skip some survey questions (farm characteristics), as the information can be added through the census data instead. Where no census data is available this information is collected via the survey.

The survey has been approved by the ETH Zürich Ethics Commission as proposal EK-2023-N-322.
