# Supplementary material for: Data on the stated willingness to accept collective agri-environmental schemes for biodiversity conservation of European grassland farmers
Source: Data Brief. 2026 Jun 17;67:112980. doi: 10.1016/j.dib.2026.112980 (PMC13315105; doi:10.1016/j.dib.2026.112980)
Supplement: Supplementary file 2 [file mmc2.pdf]

ETH
Eidgenössische Technische Hochschule Zürich
Swiss Federal Institute of Technology Zurich

Vice President for Research

ETH Zurich
Prof. Dr. Christian Wolfrum
HG F 57
Rämistrasse 101
8092 Zurich

Contact:
Office of Research
ethics@sl.ethz.ch

ETH Zürich
Dr. Robert Huber
Agrarökonomie und -politik
SOL C 9
Sonneggstrasse 33
8092 Zürich

Zurich, 16 January 2024 ZS

EK 2023-N-322: GreeNet Project: Farmer Survey and Choice Experiment on the willingness to accept collective agri-environmental schemes for biodiversity conservation on a landscape scale

Dear Mr Huber,

Your above proposal, submitted on 22 November 2023, has been reviewed by the following members of the ETH Zurich Ethics Commission:

Prof. Dr. Lutz Wingert, Präsident	Professur für Philosophie
Prof. Dr. Petra Schmid	Professur Organizational Behavior
Prof. Dr. Michael Siegrist	Institut für Umweltentscheidungen

Based on the Commission's recommendation, the Vice President for Research of ETH Zurich has come to the following decision:

☒ **Approval without reservation** ☐ Approval with reservation ☐ Revise and reply
☐ Revise and resubmit ☐ Rejection ☐ Not evaluated

Final provisions
You are required to inform the Ethics Commission on any of the following occasions:

a) if an event occurred that affects the integrity of the participants or the continuation of the research project;

b) if you wish to make changes to the research protocol or to extend the project; or

c) if the study is prematurely terminated.

Kind regards,

Christian Wolfrum Ch. Wingert

Prof. Christian Wolfrum
Vice President for Research

Prof. Lutz Wingert
Chair ETH Zurich Ethics Commission
