# Supplementary material for: Data on the stated willingness to accept collective agri-environmental schemes for biodiversity conservation of European grassland farmers
Source: Data Brief. 2026 Jun 17;67:112980. doi: 10.1016/j.dib.2026.112980 (PMC13315105; doi:10.1016/j.dib.2026.112980)
Supplement: Supplementary file 3 [file mmc3.pdf]

Eidgenössische Technische Hochschule Zürich
Swiss Federal Institute of Technology Zurich

Proposal to the ETH Zurich Ethics Commission

Project title

GreeNet Project: Farmer Survey and Choice Experiment on the willingness to accept collective agri-environmental schemes for biodiversity conservation on a landscape scale

Principal Investigator (PI)[1]

Name	Title	Group / Chair / Institute	University
Robert Huber	Dr.	Agricultural Economics and Policy Group	ETH Zurich

Involved Researchers

Name	Title	Group / Chair / Institute / Industry	University
Viviane Fahrni		Agricultural Economics and Policy Group	ETH Zurich
Robert Finger	Prof. Dr.	Agricultural Economics and Policy Group	ETH Zurich

General Information

Type of project	☒ Research ☒ PhD thesis ☒ Master thesis ☒ Bachelor thesis<br>☐ Other:
	Student applications (BA/MA): I, [*name*], confirm that my supervisor [*name*] reviewed this application ☐
Start[2]	01.01.2024
End[3]	05.2024
Method(s) of data collection (check all that apply)	☐ Interviews (☐ in person ☐ phone ☐ online)<br>☒ Survey (☐ in person ☐ phone ☒ online)<br>☐ Focus groups (☐ in person ☐ online)<br>☒ (Experimental) Behavioural study (☐ in person ☒ online)<br>☐ Social media (☐ observation ☐ intervention)<br>☐ Physiological measurements ☐ Mobile App (☐ incl. tracking)<br>☐ Photo-/video-/audio recording ☒ secondary analysis of personal data<br>☐ Student records/data[4] ☐ Other methods:

<table>
<tr><td>Number of participants</td><td colspan="2">Minimum: 100 Maximum: 400</td></tr>
<tr><td>Source(s) of funding</td><td colspan="2">Schweizerische Nationalfonds (SNF) via the European Biodiversity Partnership (Biodiversa +) in the Call BiodivProtect</td></tr>
<tr><td>Liability Insurance[5]</td><td colspan="2">☒ ETH Zurich ☐ Other:</td></tr>
<tr><td>Responsibility Kantonale Ethikkommission[6]</td><td colspan="2">☒ Not Clarified ☐ Clarified (declaration enclosed)</td></tr>
<tr><td rowspan="2">Field or lab research abroad[7]</td><td colspan="2">☐ No</td></tr>
<tr><td>☒ Yes</td><td>Countries: Germany, Austria, Norway, Estonia, Ireland
Local ethics approval: ☐ Enclosed ☒ Handed in later
☐ Not obtainable:

Comment: We hereby apply for ethical clearance for all case study regions for the survey given in the appendix. However, the AECP Group will mainly manage the survey in the Swiss case study region. Partnering countries will apply for ethical clearance with their respective institutions as well and manage their own survey rollout.</td></tr>
<tr><td rowspan="2">Clinical trial abroad</td><td colspan="2">☒ No</td></tr>
<tr><td>☐ Yes</td><td>Risk Category:
Sponsor resp. Sponsor-Investigator:
Responsible local institute:
Local PI:
Local ethics approval: ☐ Enclosed ☐ Handed in later
Registration:
Liability cover has been confirmed: ☐ Yes ☐ No</td></tr>
</table>

Proposal

1. Abstract

Agriculture is key to reach global and local biodiversity goals and governments increasingly use voluntary agri-environmental schemes to incentivize the uptake of sustainable farming practices such as extensive grassland management or creating wildflower verges. However, the currently implemented agri-environmental schemes often fail to achieve the desired environmental outcomes. One reason for their limited effectiveness is that agri-environmental schemes usually contract individual farmers and are implemented on a farm scale. However, biodiversity does not stop at farm borders, i.e. many plant and animal species require well connected landscapes to thrive. Collective agri-environmental schemes that connect farms and landscapes are seen as a way forward to make agri-environmental schemes more effective and efficient by incentivizing landscape scale management of biodiversity and ecosystem services.

The role of behavioural factors is key to increase the acceptance of collective agri-environmental schemes. Based on surveys in 6 case study regions, this project analyses the interplay of economic and behavioural factors on the willingness to participate in a collective agri-environmental scheme that aims to support biodiversity conservation on a landscape level. Participation in the study implies the filling out of an online survey including a choice experiment. Participants are asked about their personal and their farms characteristics, their risk and time preferences, environmental attitudes, attitudes on collective action, non-cognitive skills, production orientation, and social networks. Collected data will be anonymized so that individual farms cannot be back traced. No potential risks are present.

2. Project

2.1 Study Objective

The study is embedded in the inter- and transdisciplinary research project "Grassland conservation across European landscapes protecting biodiversity and ecosystem services with ecological networks" (GreeNet) financed by the Swiss National Science Foundation via the European Biodiversity Partnership (Biodiversa +) in the Call BiodivProtect. The project comprises research groups from Austria, Germany, Norway, Ireland, Estonia, and Switzerland. In this particular study we elicit farmer's willingness to accept policy schemes that incentivize increased collective action (on a range from cooperation to collaboration) for biodiversity conservation in grassland production, as well as understanding which behavioural factors influence the uptake of such schemes.

2.2 Methods and Study Design

For all case studies data will be collected through an online survey including a choice experiment. The survey will be implemented via LimeSurvey LimeSurvey — Free Online Survey Tool. *In Switzerland LimeSurvey is run on our own ETH Zürich survey server ensuring that data at all stages remains solely at ETH Zürich. Partnering countries store their own data on their respective servers.*
The survey will be rolled out in the Swiss case study region among all 2'179 farms (as of 2021) located in the Canton of Grisons. Farmers will receive an e-mail with an invitation to the LimeSurvey questionnaire. E-mail addresses were provided by the Canton of Grisons (see data use contract with the canton of Grisons, separate document). Filling out the survey takes about 30 minutes.
The survey is available in German and Italian for the Swiss case study region. Partnering countries translate the survey to their respective languages. The survey consists of a set of questions and a discrete choice experiment. Farmers are asked about their personal and their farms characteristics, their risk and time preferences, environmental attitudes, attitudes on collective action, non-cognitive skills, production orientation, and social networks.
In the choice experiment participants are asked to choose between different policy schemes which differ with regards to several attributes. The choice experiment consists of ten choice cards, which each hold three alternative options (A, B and C) to choose from. The two alternatives A and B differ with regards to four attributes (Partner farmers in collective, Monitoring, Discretion, and Additional Payment). Option C represents an opt-out, for when neither A nor B are acceptable options for survey participants.
The full survey including the choice experiment can be found in the Appendix.
There is no necessary or incomplete disclosure of information or deception of participants.
Survey participants are asked for their consent before filling out the survey. In the Swiss case study region the consent form is available in German and Italian. Partnering countries translate the consent form to their respective languages.

2.3 Participants

The survey will be performed in each project partner country's respective case study region (see 2.5 Project Partners and Funding). The case study regions have been chosen to represent a gradient of grassland conservation states.
In Switzerland, we will contact farmers living in the Canton of Grisons for participation in the online survey via Limesurvey through a cantonal database, which we received by the canton of Grisons. Participation in the survey is voluntary and can be withdrawn at any time. There are 2'179 farms in the canton of Grisons (2021) to whom the survey can be sent. We expect a response rate of 10-15% and aim for a sample size exceeding 100 observations.
In the Swiss case study people can participate in the survey and choice experiment if they are on the E-mail list provided by the Canton of Grisons. This list includes all farmers of the Canton of Grisons. The goal is to elicit the opinions and preferences of farmers who focus on grassland production. Since grassland production is prevalent in the Canton of Grisons (94% of farmland) all contacted farmers qualify for the survey.

2.4 Project Schedule

Date	Project phase	Description
04.2023	Literature review / research	Search for state-of-the-art literature on collective action for specifically biodiversity conservation and more generally agri-environmental schemes, as well as literature on choice experiment design
06.2023 – 09.2023	Survey design / feedback	Construct survey and get feedback from the GreeNet consortium and the projects' scientific advisory board.
12.2023	Pre-test / pilot	Pre-testing of the survey with young farmers (e.g. via students from the Strickhof)
12.2023	Survey update	Update / reformulation of the survey design based on the feedback from pre-tests / pilot.
02.2024 – 06.2024	Roll-out	The survey is sent online to participants via email.
07.2024	Analyses	Conducting analyses and generating a report for the participants who requested feedback.
2025	Publication of results	Scientific paper writing and publication in academic journals. A summary of results for stakeholders in policy and industry will be published. Moreover, a short feedback report in German and Italian will be sent to participants (if they want) and involved cantonal agencies.

2.5 Project Partners and Funding

The study is embedded in the inter- and transdisciplinary research project "Grassland conservation across European landscapes protecting biodiversity and ecosystem services with ecological networks" (GreeNet) financed by the Swiss National Science Foundation via the European Biodiversity Partnership (Biodiversa +) in the Call BiodivProtect. The project comprises research groups from Austria, Germany, Norway, Ireland, Estonia, and Switzerland. Project partners include:

- Department of Economics and Social Sciences – Institute of Sustainable Economic Development, University of Natural Resources and Life Sciences BOKU, Vienna, Austria
- Department of Botany and Biodiversity Research – Biodiversity Dynamics and Conservation, University Vienna, Vienna, Austria
- Farm economics and ecosystem services (Working groups "Farm economics and ecosystem services" and "Provisioning of Biodiversity"), Leibniz Centre for Agricultural Landscape Research ZALF e. V., Müncheberg, Germany

- Institute of Agricultural and Environmental Sciences, Estonian University of Life Sciences EMÜ, Tartu, Estonia
- Applied Ecology Unit – School of Natural Sciences SNS, University of Galway, Galway, Ireland
- Norwegian Institute for Nature Research NINA, Oslo, Norway
- Research Department, Ruralis Institute for Rural and Regional Research, Oslo, Norway
- Agriculture and Biodiversity Group, Federal Office of Agriculture – Agroscope, Affoltern, Switzerland
- Agricultural Economics and Policy Group, Eidgenössische Technische Hochschule ETH Zurich (Swiss Federal Institute of Technology), Zurich, Switzerland

In addition, the GreeNet project features a stakeholder and a scientific advisory board, consisting of external advisors from research, industry, policy, and practice. Their role involves the provision of feedback, the access to networks, but not the steering or influencing of the experiment or analysis of the collected data. A wide range of stakeholders is informed and involved in dialogues also about this survey via the project communication at large.
The survey is a collaborative endeavor. Project partners in each country (CH, DE, AT, IE, EE, NO) are responsible for their own implementation of the survey. Responsibility for leading the survey development lays with AECP ETHZ. Active feedback is contributed by all project partners. Project partners have to verify the relevance and coherence of the survey questions for their respective case study regions and are responsible for providing a translation. If a project partner country can not implement the survey themselves due to restricted resources (such as for example having no economics team in the project, only one on ecology) AECP offers to take over implementation in LimeSurvey. However, the project partners are responsible for the distribution of the link to the survey in their case study region. In this case the collected data will be handed over to the respective project partners after the survey has been closed. Both individual papers on each case study region as well as a joint paper on overall findings are in planning. For this all contributing authors need access to the anonymized data from all project partners. All project partners need to apply for ethical clearance with their respective ethics institution regardless of which project partner hosts the respective data collection.

3. Ethical Aspects

3.1 Informed Consent and Debriefing

In Switzerland participants are contacted via e-mail and invited to the LimeSurvey link.
Participants find detailed study information in the introduction and the data protection terms at the beginning of the survey. They are informed that their survey response will be linked with a secondary data set we received from the Canton of Grisons. This data set includes spatially explicit farm information on participation in biodiversity conservation schemes as well as farm structural data, and personal data (phone number, address, and e-mail address).
Participants are asked for their consent after reading the introduction and the data protection terms and before answering any questions in the survey. Participation in the survey is voluntary and can be withdrawn at any time. Participants can stop filling out the survey at any time. After filling out the survey participants can contact AECP to withdraw their answers at any time.
Consent forms are stored together with the data on secure ETH servers. We envision permanent storage of data and documentation at ETH servers.
As no incomplete information or deception is part of the study design, debriefing mainly consists of sending interested participants a summary report of the survey results and of answering any questions participants may send in via e-mail.
Study results will be made available to interested participants in the form of a summary report. This report will be produced and distributed within 2-3 months after the survey has been closed. In addition, we will write blog posts in German (https://agrarpolitik-blog.com/) based on publications from this data and share it with the canton and involved stakeholders.

3.2 Data Protection and Publication

In the Swiss case study data is collected as personal data, since names and e-mail addresses are made available to AECP by the Canton of Grisons, and participants are contacted directly through this information which is then stored together with the survey results in LimeSurvey. For further use and publication the data is then anonymized. Other case study regions may use the same approach where e-mail addresses are available.
The data collected through the survey needs to be personal (e.g. including an e-mail address) in order to link it to other data sets provided to AECP by the Canton of Grisons, which also contain personal data (e.g. including e-mail address, name, etc.). Other personal data collected in the survey like behavioural factors and farmer's characteristics (age, gender, education, farm succession) and preferences are key to understanding farmer's decision making.
After linking the data sets farm identifying attributes (e.g. e-mail addresses or combinations of attributes) are separated from the main file, here referred to as master. The master is linked through a unique identifier to the main dataset and stored in a password-protected file. The dataset and master are stored and archived on secure servers at ETH Zurich. Access to the master is restricted to the principal applicant and involved researchers only. The data is anonymized before it is archived in ETH Zürich long-term storage facilities (e.g. ETH Research Collection).
All data is completely anonymised in accordance with ETH Law Art. 36d.
To guarantee data security and protection of the personal data, appropriate data use agreements have been drawn between the project partners.
Results are published in anonymized form and will be made accessible in a repository.

3.3 Compensation

In the Swiss case study we run a lottery of 100 Landi vouchers valued at 30 CHF each among the interested participants.

3.4 Risks and Countermeasures

The survey results may have an influence on the agricultural politics debate in Switzerland but no physical and psychological risks and discomforts are to be expected for the participants. Therefore no specific countermeasures are foreseen. Nevertheless, the publication(s) of the study results will be carefully prepared with stakeholders and farmers, and embedded in the overarching GreeNet project.

3.5 Risk-Benefit Analysis

There are little to no risks associated with this study. The results of the study help inform policymakers how to design policies that encourage practices which will increase collective action and biodiversity levels in Swiss agriculture.

4. References

None.

Explanatory notes

[1] The (co-)principal investigator (PI) must be affiliated with ETH Zurich. The PI is usually a professor or a senior scientist, or the supervising person in the case of Bachelor or Master theses (students are listed as "involved researchers").

[2] The start and end dates should refer to the human subject research part of the entire project. This part may only be started once the application has been reviewed by the Ethics Commission and approved by the Vice President for Research (this also applies to the recruitment of participants). The complete approval process can take up to eight weeks, which is why the requested start date should be at least eight weeks in the future (however, the project may be started once the approval is issued). Student projects are reviewed in a shortened procedure. For more information, see "How long it takes".

[3] Extensions can be applied for as an amendment in Etappo (tab "Amendments"; see also "Amendments").

[4] Please refer to the guidelines "Educational Research".

[5] Adverse health effects that are directly caused by participating in the study and can be demonstrated to be attributable to fault on the part of the project team or ETH Zurich are covered by ETH's liability insurance, exclusively an excess of CHF 1500 payable by the chair (cf. "Insurance").

[6] *Biomedical Human Subject Research* is regulated by the Human Research Act (HRA) and corresponding ordinances (ClinO, HRO, TPA, etc.). Research involving one or more human subjects, undertaken to systematically assess the safety or performance of medical devices are deemed to be *clinical trials of medical devices,* where the HRA and the Ordinance on Clinical Trials with Medical Devices (ClinO-MD) define the corresponding requirements. Both types of research must be approved by a Kantonale Ethikkommission and, if necessary, by Swissmedic. In case of doubt, contact the responsible Kantonale Ethikkommission or obtain a clarification of responsibility. If your project is approved by a Kantonale Ethikkommission, no additional review by the ETH Zurich Ethics Commission is necessary.

[7] Whenever possible, field research abroad must also be approved by a local ethics committee (e.g., of an institute of a partner). If the research is to be conducted in a risk area, it must also be clarified with the Safety, Security, Health and Environment (SSHE) department before submitting the ethics application whether a safety concept must be submitted.

Appendix A

Eidgenössische Technische Hochschule Zürich
Swiss Federal Institute of Technology Zurich

Information and consent form (Will be provided online to participants and consent is given by ticking a particular answer box. CH: Will be provided in German and Italian.)

GreeNet Project: Farmer Survey and Choice Experiment on the willingness to accept collective agri-environmental schemes for biodiversity conservation on a landscape scale

Participant (full name):	………………………………………………….
Contact person (full name):	Robert Huber (rhuber@ethz.ch)<br>Agrarökonomie- und Politik, ETH Zürich
Conducting person:	Viviane Fahrni (vfahrn@ethz.ch)<br>Robert Finger (rofinger@ethz.ch)<br>Agrarökonomie- und Politik, ETH Zürich
Data Protection Officer ETH Zurich:	Tomislav Mitar (tomislav.mitar@sl.ethz.ch)

We would like to ask you if you are willing to participate in our research project. Your participation is voluntary. Please read the text below carefully and ask the conducting person about anything you do not understand or would like to know.

What is investigated and how?
The objective of the study, that is part of a Swiss National Science Foundation (SNF) funded project and done in collaboration with the Canton of Grisons, is to assess the influence of different behavioural factors on farmers production decisions related to biodiversity conservation in grasslands in order to design agricultural policies that enable the reconciliation of biodiversity conservation and food production and secure a sustainable landscape management.

Who can participate?
The survey is aimed at all farmers in the Canton of Grisons.

What am I supposed to do as a participant?
As a participant you will answer questions regarding yourself and your farm's characteristics, as well as questions regarding your preferences and attitudes for risk management, time, social network, cooperation, environment, trust and production orientation. You will also fill out a choice experiment which means that you will be repeatedly asked to choose between three hypothetical policy options.

What are my rights during participation?
Your participation in this study is voluntary. You may stop filling out the survey and close the browser at any time. You may withdraw your participation at any time without specifying reasons and without any disadvantages. You may contact the AECP group at any time after having filled out the survey to withdraw your responses.

What risks and benefits can I expect?
There are no risks involved. Data will be treated anonymously, and no sensitive information is shared with third parties. At the end of the project, the cantons, farms associations and farmers (if requested) will receive a report with a summary of the results.

Will I be compensated for participating?
If participants are interested, they can participate in a lottery of 100 Landi vouchers (30 CHF each). (Neither Landi, nor Fenaco, nor any other institutions are involved in this study.)

What data is collected from me and how is it used?
In the survey data on preferences towards agricultural practices and some farm information will be collected.

Namely:
- Personal characteristics (age, gender, education, farm succession)
- Farm characteristics
- Preferences in the choice experiment
- Attitudes towards cooperation
- Attitudes towards the environment
- Attitudes towards self-efficacy
- Risk and time preferences
- Attitudes towards trust
- Social network preferences
- Income satisfaction and production orientation
- Your comments

Additionally, if you complete the questionnaire, your responses will be linked to a secondary data set given to AECP by the Canton of Grisons for the purpose of this study. This data set includes spatially explicit farm information on participation in biodiversity conservation schemes as well as farm structural data, and personal data (your phone number, address, and e-mail address).
Personal data such as your e-mail address is linked to your survey responses in order for us to send out summary reports and the Landi vouchers (to interested participants). This personal data will be removed from the dataset before the data is used for scientific purposes.
Members of the ETH Zurich Ethics Commission may access the original data for examination purposes. Strict confidentiality will be observed at any time. All identifying data will be deleted and all other data anonymised as soon as the purpose of the data processing allows this. Data will only be published in anonymized form so that no conclusions can be drawn about individuals.
Anonymised data will be stored in a repository (ETH Research Collection). After 10 years, the data will be moved to the ETH Archive for a long-term preservation of data and the reproducibility of the results.

What are my rights to my personal data?

Before the irrevocable anonymization of the collected data, you can request information about the personal data collected from you at any time and without giving reasons. You can also request that it be rectified, handed over to you, barred for processing or erased. To do so, please contact the person indicated above.

Who funds this study?
This study is funded by the Swiss National Science Foundation (SNF).

Who reviewed this study?
This study was examined by the ETH Zurich Ethics Commission as proposal *EK-2023-N-322-R.*

Complaints office
The secretariat of the ETH Zurich Ethics Committee is available to help you with complaints in connection with your participation. Contact: *ethics@sl.ethz.ch* or 0041 44 632 85 72.

Consent Form

I, the participant, confirm by my ticking the box given below that:

- I have read and understood the study information. My questions have been answered completely and to my satisfaction.
- I comply with the inclusion and exclusion criteria for participation described above. I am aware of the requirements and restrictions to be observed during the study.
- I have had enough time to decide about my participation.
- I participate in this study voluntarily and consent that my personal data be used as described above.
- I understand that I can stop participating at any moment.

I would like to be informed about the results of this study

☐ Yes
☐ No

Appendix B-n: Instruments (surveys, interview questions, etc.)

GreeNet Farmer Survey

Dear farmers,

within an international project (GreeNet) we, from the Agricultural Economics and Policy group at ETH Zurich, investigate possibilities for biodiversity conservation on a landscape scale in Switzerland. Within this survey we would like to learn which factors influence your farm management decisions and potential cooperation with other farmers. The survey is funded by the Swiss National Science Foundation (SNSF).

We are dealing with the options of biodiversity conservation in Swiss agriculture. In particular, we are interested in better understanding farmers' decision-making with regards to biodiversity conservation measures and collaboration with other farmers in systems where they can plan and implement measures self-determinedly and according to their circumstances. Your assessments and personal network information, preferences and risk attitudes are central to this. Since you as a farmer in Switzerland have experience with instruments such as the Biodiversity payments (BFF Q1 and Q2), Network payments and Landscape quality payments, we are especially interested to learn how to improve such concepts from your evaluation. The results of the survey will provide important insights for agricultural practice, advice, and research.

The survey consists of three sections: 1) questions regarding you and your farm, 2) a Choice Experiment, and 3) questions about your personal perception and preferences. Completing the questionnaire takes about 20-30 minutes.

If you are interested, we will gladly send you a summary of the survey results. Moreover, we are also raffling 100 Landi vouchers worth 30 CHF each among the survey participants. Your data and information will be kept strictly confidential and will be used anonymously for scientific purposes only.

We thank you very much for your participation!

For questions, please contact:

Viviane Fahrni,

Agricultural Economics and Policy Group (AECP), ETH Zurich

vfahrni@ethz.ch

Best regards,

Viviane Fahrni (ETH Zurich)

There are 87 questions in this survey.

Consent and Administration

Your participation in the survey is voluntary. Your data and information will of course be treated with strict confidentiality and used anonymously for scientific purposes. Please agree to the information and the conditions of participation and data protection that can be found here [LINK]

*

Please choose **only one** of the following:

◯ I hereby confirm that my participation is voluntary and that my data may be used.

Would you like to receive the results of the survey? We will send you a summary of the results.

*

Please choose **only one** of the following:

- Yes
- No

Would you like to participate in the lottery of 100 Landi vouchers valued at CHF 30 each? (Neither Landi, nor Fenaco, nor any other instituion are involved in this study)

*

Please choose **only one** of the following:

- Yes
- No

Farmer or farm manager characteristics

What is your gender? *

Please choose **only one** of the following:

- Male
- Female
- Non-binary
- Do not want to say

How old are you?

*

Please choose **only one** of the following:

- 18-25
- 26-35
- 36-45
- 46-55
- 56-65
- 66 or older

What year did you take over the business?

Please write your answer here:

What is the highest education that you have completed? *

Please choose **only one** of the following:

- ◯ Agricultural practician EBA or Farmer EFZ
- ◯ Agricultural management school / “Meister” or “Betriebsleiter” degree / Agricultural business school
- ◯ Higher technical school (HF) (agricultural technician), University of applied sciences (BSs/MSc) or University/ETH
- ◯ Other

If you chose "Other", please specify: *

Only answer this question if the following conditions are met:
Answer was 'Other' at question ' [Education]' (What is the highest education that you have completed?)

Please write your answer here:

Do you have a successor to your farm? *

Please choose **only one** of the following:

- ◯ Yes
- ◯ No
- ◯ Not yet relevant

Farm and farm characteristics

What is your farm size in hectares? (Including all areas, arable, grasslands, etc.) *

Please write your answer here:

What is your postal code? *

Please write your answer here:

What’s the number of people working in your business in full-time equivalents? (including family workers)

*

Please write your answer here:

What share of land are you leasing?

*

Please choose **only one** of the following:

- 0-25%
- 26-50%
- 51-75%
- 76-100%

According to which production form do you manage your farm?

*

Please choose **only one** of the following:

- Conventional
- Ecological performance assessment (ÖLN)
- Integrated production (IP-Suisse)
- Organic or Demeter (Bio-dynamic)
- Other

Which of these direct payment programs does your farm participate in?

*

Please choose **all** that apply:

- ☐ None
- ☐ BFF Q1 (including the mandatory 7% for cross compliance)
- ☐ BFF Q2
- ☐ Network Bonus
- ☐ Landscape quality project

How much of your area is contracted under these direct payment schemes (combined)? *

Only answer this question if the following conditions are met:
Answer was 'BFF Q1 (including the mandatory 7% for cross compliance)' *or* 'Landscape quality project' *or* 'BFF Q2' *or* 'Network Bonus' at question ' [PaymentSchemes]' (Which of these direct payment programs does your farm participate in?)

Please choose **only one** of the following:

- ◯ 1% - 10%
- ◯ 11% - 20%
- ◯ 21% - 30%
- ◯ 31% - 40%
- ◯ 41% - 50%
- ◯ 51% - 60%
- ◯ 61% - 70%
- ◯ 71% - 80%
- ◯ 81% - 90%
- ◯ 91% - 100%

Is your farm located in an area with protection status, and if yes which?

*

Please choose **all** that apply:

- ☐ No
- ☐ Yes, Nature reserve
- ☐ Yes, UNESCO-Biosphere
- ☐ Yes, Smaragd area
- ☐ Yes, Regional Nature Park
- ☐ Yes, Forest reserve
- ☐ Yes, other

If you chose "Yes,other", in which? *

Only answer this question if the following conditions are met:
Answer was at question ' [ProtectionArea]' (Is your farm located in an area with protection status, and if yes which?)

Please write your answer here:

Choice Experiment Introduction

Description of the Choice Cards

The following section is about evaluating different combinations of collaboration for the promotion of biodiversity. You will be asked to **choose between three options**. The first two options (A and B) represent scenarios of collaboration in biodiversity conservation schemes. **Imagine that these options (A or B) replace any agri-environmental biodiversity conservation schemes which you may currently implement on your farm**. These options differ with regards to four characteristics, which will be explained in more detail in the table below. **The third option (C) represents a situation in which you chose none of the two biodiversity conservation schemes (A and B) and you continue to manage your farmland as you do today.**

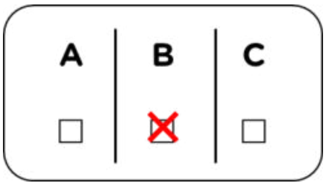

Figure 1: Schematic representation of a choice card

Compare the three options and reflect **which combination of characteristics agree with you more.** If you prefer one option mark it by clicking the checkbox below it. **If none of the two options seem reasonable for you and your farm, choose checkbox C ("none of the two").** For us to gather information about the different characteristics you are asked to choose a **total of 10 times.** For every choice set indicate your preferred option and then click "continue".

*

A more extensive **explanation** can be found in the **text and video on the next page**. Both are available to you during the entire choice experiment.

Please consider that the **choice cards represent a hypothetical scenario**. Studies show that **people tend to act differently when they face hypothetical decisions**, for example because the true implications are hard to estimate. **Please fill out the choice cards truthfully and to the best of your knowledge, as if the scheme were to be truly implemented on your farm.**

Are you ready to continue?

*

Please choose **all** that apply:

☐ Yes

Choice Experiment Description

Description of the basis

Many plant and animal species benefit from well-connected habitats over an entire landscape. The goal of the hypothetical agri-environmental scheme is therefore to **support biodiversity not just on single farms but in the whole region and on a landscape scale**. For this state and regional institutions define biodiversity targets. These targets can be measured according to indicator plants and animals. A target is considered as reached when a certain number of indicator plants and animals can be found on a unit of land.

If you enter the policy scheme, you are guaranteed that the contract will last for at least 5 years at a time.

Description of the Options

Attributes	Description	Possible valu[illegible]
**Collective with**	To achieve the biodiversity targets, you are asked to work collectively with other farmers in the biodiversity conservation scheme. Specifically, you can build a group with **one other farmer, or with 3, 6, 10 or 20 other farmers**. Group members should be managing land in the same region, ideally as close to each other as possible, but it is not necessary that they are direct neighbors. If you are in such a group for one of the conservation schemes, the whole group works together to define the measures and where these are to be implemented. How exactly the group is organized and how members work together is up to the group members.	**1, 3, 6, 10 [illegible]** farm[illegible]
**Discretion**	To achieve the biodiversity targets, several measures are possible. For example, extensive management such as reduced fertilization or late mowing, or any measure that is meaningful in your region could be used.<br><br>In the conservation schemes there are two ways to choose the measures:<br><br>1. **A consultant from the canton defines** a plan and sends a project description and a contract to the canton. The farmers in the collective do not have to do the planning work for themselves, but also do not have the freedom to choose where to implement which measure.<br><br>2. **The farmers in the group decide** for themselves which measures to implement and send a project description and a contract to the canton. This requires work for planning and coordinating but the farmers collective has more freedom to choose where to implement which measure.<br><br>In both cases the farmers collective can receive general consultation from cantonal extension services. The difference of the two cases is in **who does the planning and the writing of the project description.**	(1) a consulta[illegible] **canton** defin[illegible]<br><br>(2) **the farmer[illegible]** dec[illegible]
**Monitoring**	To check whether the biodiversity targets have been reached there are two monitoring options:<br><br>1. An **expert** evaluates the results with an **in-person** visit of the landscape, including your farm. This requires some of your time and you get to interact with the expert. The expert can only focus on **indicator plant species on a cross section** of the field and the result is therefore less representative of the actual biodiversity.<br><br>2. **Data** of the farmland is collected by a combination of **digital tools**. You do not need to spend your time on the evaluation, but you also don't get to interact with an expert. The data collected by modern technology considers the **whole field** and can **additionally model** insect and bird populations and the result is therefore more representative of the actual biodiversity.	(1) expert [illegible]<br><br>(2) **digit[illegible]**
**Additional Payment**	For collectively reaching the biodiversity targets you are compensated. Since the biodiversity targets are more ambitious than what is done today, **your group would receive these payments in addition to what you receive for your biodiversity conservation efforts today**. Depending on the conservation scheme your group receives an additional **500, 1000 or 1500 CHF per hectare on which the targets have been reached**. This money is paid to the group and can be distributed freely among the members so that their individual contributions are compensated fairly. For partial reaching of targets there will be a partial reduction in payments.	an additional [illegible] **1500 CHF** pe[illegible] year on whic[illegible] have bee[illegible]

[LINK VIDEO]

Choice Experiment - Choice Set 1

Choice Set 1

	**Option A**	**Option B**	**None of the two**
**Additional Payments** compared to current levels	+1500 CHF per ha and year	+500 CHF per ha and year	No change
**Collective with** number of people you are in a group with	10	3	No change
**Monitoring** in person or via digital tools	Digital tools	Digital tools	No change
**Discretion** with the local government or farmers collective	State	State	No change
**Your Choice**			

*

Please choose the appropriate response for each item:

◯	◯	◯

Please indicate on how much more or less of your land (in percentage) you would be willing to implement biodiversity conservation measures under the chosen scheme. *

Only answer this question if the following conditions are met:
Answer was '' *or* '' at question ' [CECS1]' (Choice Set 1 Option A Option B None of the two Additional Payments compared to current levels +1500 CHF per ha and year +500 CHF per ha and year No change Collective with number of people you are in a group with 10 3 No change Monitoring in person or via digital tools Digital tools Digital tools No change Discretion with the local government or farmers collective State State No change Your Choice $(document).on('ready pjax:scriptcomplete',function(){ // Identify this question var thisQuestion = $('#question{QID}'); // Move the radios $('.question-text table:eq(0) tr:last td:eq(2)', thisQuestion).append($('.subquestion-list .answers-list:eq(0) .answer-item:eq(0) *', thisQuestion)); $('.question-text table:eq(0) tr:last td:eq(4)', thisQuestion).append($('.subquestion-list .answers-list:eq(0) .answer-item:eq(1) *', thisQuestion)); $('.question-text table:eq(0) tr:last td:eq(6)', thisQuestion).append($('.subquestion-list .answers-list:eq(0) .answer-item:eq(2) *', thisQuestion)); // Some classes for presentation $('.question-text table:eq(0) input:radio', thisQuestion).closest('td').addClass('answer-item radio-item text-center radio'); $('.question-text table:eq(0) .radio-item label', thisQuestion).show(); // Click event on the table cells $('.question-text table:eq(0) .radio-item', thisQuestion).on('click', function(e) { $('input:radio', this).trigger('click'); }); $('.question-text table:eq(0) input:radio', thisQuestion).on('click', function(e) { e.stopPropagation(); }); // Clean-up styles $('.answer-container', thisQuestion).hide(); $('.question-text table:eq(0) .label-text', thisQuestion).remove(); $('.question-text table:eq(0) .radio-text', thisQuestion).css({ 'cursor': 'pointer' }); }); ())

Please choose **only one** of the following:

- ◯ -10% or less
- ◯ 0 to -10%
- ◯ 0% (no change)
- ◯ 0 to +10%
- ◯ +10% or more

Helpdesk - Click here to see the explanations again:

Please choose **only one** of the following:

- ◯ Explanatory Video
- ◯ Explanatory Text

{CEDescriptionBasis.question}

{CEDescriptionOptions.question}

Only answer this question if the following conditions are met:
Answer was 'Explanatory Text' at question ' [CECS1Helpdesk]' (Helpdesk - Click here to see the explanations again: $(document).on('ready pjax:scriptcomplete',function(){ $('.button-item:contains("No answer") label').text('Hide Help'); });)

Choice Experiment Choice Set 2

Choice Set 2

	**Option A**	**Option B**	**None of the two**
**Additional Payments** compared to current levels	+1500 CHF per ha and year	+500 CHF per ha and year	No change
**Collective with** number of people you are in a group with	10	3	No change
**Monitoring** in person or via digital tools	Digital tools	Digital tools	No change
**Discretion** with the local government or farmers collective	State	State	No change
**Your Choice**			

*

Please choose the appropriate response for each item:

	◯	◯	◯

{CECS1Area.question}

*

Only answer this question if the following conditions are met:
Answer was '' *or* '' at question ' [CECS2]' (Choice Set 2 Option A Option B None of the two Additional Payments compared to current levels +1500 CHF per ha and year +500 CHF per ha and year No change Collective with number of people you are in a group with 10 3 No change Monitoring in person or via digital tools Digital tools Digital tools No change Discretion with the local government or farmers collective State State No change Your Choice $(document).on('ready pjax:scriptcomplete',function(){ // Identify this question var thisQuestion = $('#question{QID}'); // Move the radios $('.question-text table:eq(0) tr:last td:eq(2)', thisQuestion).append($('.subquestion-list .answers-list:eq(0) .answer-item:eq(0) *', thisQuestion)); $('.question-text table:eq(0) tr:last td:eq(4)', thisQuestion).append($('.subquestion-list .answers-list:eq(0) .answer-item:eq(1) *', thisQuestion)); $('.question-text table:eq(0) tr:last td:eq(6)', thisQuestion).append($('.subquestion-list .answers-list:eq(0) .answer-item:eq(2) *', thisQuestion)); // Some classes for presentation $('.question-text table:eq(0) input:radio', thisQuestion).closest('td').addClass('answer-item radio-item text-center radio'); $('.question-text table:eq(0) .radio-item label', thisQuestion).show(); // Click event on the table cells $('.question-text table:eq(0) .radio-item', thisQuestion).on('click', function(e) { $('input:radio', this).trigger('click'); }); $('.question-text table:eq(0) input:radio', thisQuestion).on('click', function(e) { e.stopPropagation(); }); // Clean-up styles $('.answer-container', thisQuestion).hide(); $('.question-text table:eq(0) .label-text', thisQuestion).remove(); $('.question-text table:eq(0) .radio-text', thisQuestion).css({ 'cursor': 'pointer' }); }); ())

Please choose **only one** of the following:

- ◯ -10% or less
- ◯ 0 to -10%
- ◯ 0% (no change)
- ◯ 0 to +10%
- ◯ +10% or more

Helpdesk - Click here to see the explanations again:

Please choose **only one** of the following:

- ◯ Explanatory Video
- ◯ Explanatory Text

{CEDescriptionBasis.question}

{CEDescriptionOptions.question}

Only answer this question if the following conditions are met:
Answer was 'Explanatory Text' at question ' [CECS2Helpdesk]' (Helpdesk - Click here to see the explanations again: $(document).on('ready pjax:scriptcomplete',function(){ $('.button-item:contains("No answer") label').text('Hide Help'); });)

Choice Experiment - Choice Set 3

Choice Set 3

	**Option A**	**Option B**	**None of the two**
**Additional Payments** compared to current levels	+1000 CHF per ha and year	+500 CHF per ha and year	No change
**Collective with** number of people you are in a group with	1	3	No change
**Monitoring** in person or via digital tools	In person	In person	No change
**Discretion** with the local government or farmers collective	Farmers	Farmers	No change
**Your Choice**			

*

Please choose the appropriate response for each item:

	○	○	○

{CECS1Area.question}

*

Only answer this question if the following conditions are met:
Answer was '' *or* '' at question ' [CECS3]' (Choice Set 3 Option A Option B None of the two Additional Payments compared to current levels +1000 CHF per ha and year +500 CHF per ha and year No change Collective with number of people you are in a group with 1 3 No change Monitoring in person or via digital tools In person In person No change Discretion with the local government or farmers collective Farmers Farmers No change Your Choice $(document).on('ready pjax:scriptcomplete',function(){ // Identify this question var thisQuestion = $('#question{QID}'); // Move the radios $('.question-text table:eq(0) tr:last td:eq(2)', thisQuestion).append($('.subquestion-list .answers-list:eq(0) .answer-item:eq(0) *', thisQuestion)); $('.question-text table:eq(0) tr:last td:eq(4)', thisQuestion).append($('.subquestion-list .answers-list:eq(0) .answer-item:eq(1) *', thisQuestion)); $('.question-text table:eq(0) tr:last td:eq(6)', thisQuestion).append($('.subquestion-list .answers-list:eq(0) .answer-item:eq(2) *', thisQuestion)); // Some classes for presentation $('.question-text table:eq(0) input:radio', thisQuestion).closest('td').addClass('answer-item radio-item text-center radio'); $('.question-text table:eq(0) .radio-item label', thisQuestion).show(); // Click event on the table cells $('.question-text table:eq(0) .radio-item', thisQuestion).on('click', function(e) { $('input:radio', this).trigger('click'); }); $('.question-text table:eq(0) input:radio', thisQuestion).on('click', function(e) { e.stopPropagation(); }); // Clean-up styles $('.answer-container', thisQuestion).hide(); $('.question-text table:eq(0) .label-text', thisQuestion).remove(); $('.question-text table:eq(0) .radio-text', thisQuestion).css({ 'cursor': 'pointer' }); }); ())

Please choose **only one** of the following:

- ◯ -10% or less
- ◯ 0 to -10%
- ◯ 0% (no change)
- ◯ 0 to +10%
- ◯ +10% or more

Helpdesk - Click here to see the explanations again:

Please choose **only one** of the following:

- ◯ Explanatory Video
- ◯ Explanatory Text

{CEDescriptionBasis.question}

{CEDescriptionOptions.question}

Only answer this question if the following conditions are met:
Answer was 'Explanatory Text' at question ' [CECS3Helpdesk]' (Helpdesk - Click here to see the explanations again: $(document).on('ready pjax:scriptcomplete',function(){ $('.button-item:contains("No answer") label').text('Hide Help'); });)

Choice Experiment - Choice Set 4

Choice Set 4

	Option A	Option B	None of the two
**Additional Payments** compared to current levels	+500 CHF per ha and year	+1000 CHF per ha and year	No change
**Collective with** number of people you are in a group with	6	3	No change
**Monitoring** In person or via digital tools	In person	In person	No change
**Discretion** With the local government or farmers collective	State	State	No change
**Your Choice**			

*

Please choose the appropriate response for each item:

	○	○	○

{CECS1Area.question}

*

Only answer this question if the following conditions are met:
Answer was '' *or* '' at question ' [CECS4]' (Choice Set 4 Option A Option B None of the two Additional Payments compared to current levels +500 CHF per ha and year +1000 CHF per ha and year No change Collective with number of people you are in a group with 6 3 No change Monitoring In person or via digital tools In person In person No change Discretion With the local government or farmers collective State State No change Your Choice $(document).on('ready pjax:scriptcomplete',function(){ // Identify this question var thisQuestion = $('#question{QID}'); // Move the radios $('.question-text table:eq(0) tr:last td:eq(2)', thisQuestion).append($('.subquestion-list .answers-list:eq(0) .answer-item:eq(0) *', thisQuestion)); $('.question-text table:eq(0) tr:last td:eq(4)', thisQuestion).append($('.subquestion-list .answers-list:eq(0) .answer-item:eq(1) *', thisQuestion)); $('.question-text table:eq(0) tr:last td:eq(6)', thisQuestion).append($('.subquestion-list .answers-list:eq(0) .answer-item:eq(2) *', thisQuestion)); // Some classes for presentation $('.question-text table:eq(0) input:radio', thisQuestion).closest('td').addClass('answer-item radio-item text-center radio'); $('.question-text table:eq(0) .radio-item label', thisQuestion).show(); // Click event on the table cells $('.question-text table:eq(0) .radio-item', thisQuestion).on('click', function(e) { $('input:radio', this).trigger('click'); }); $('.question-text table:eq(0) input:radio', thisQuestion).on('click', function(e) { e.stopPropagation(); }); // Clean-up styles $('.answer-container', thisQuestion).hide(); $('.question-text table:eq(0) .label-text', thisQuestion).remove(); $('.question-text table:eq(0) .radio-text', thisQuestion).css({ 'cursor': 'pointer' }); }); ())

Please choose **only one** of the following:

- ◯ -10% or less
- ◯ 0 to -10%
- ◯ 0% (no change)
- ◯ 0 to +10%
- ◯ +10% or more

{CECS1Helpdesk.question}

Please choose **only one** of the following:

- ◯ Explanatory Video
- ◯ Explanatory Text

{CEDescriptionBasis.question}

{CEDescriptionOptions.question}

Only answer this question if the following conditions are met:
Answer was 'Explanatory Text' at question ' [CECS4Helpdesk]' ({CECS1Helpdesk.question} $(document).on('ready pjax:scriptcomplete',function(){ $('.button-item:contains("No answer") label').text('Hide Help'); });)

Choice Experiment - Choice Set 5

Choice Set 5

	Option A	Option B	None of the two
**Additional Payments** compared to current levels	+500 CHF per ha and year	+1000 CHF per ha and year	No change
**Collective with** number of people you are in a group with	10	15	No change
**Monitoring** in person or via digital tools	In person	Digital tools	No change
**Discretion** with the local government or farmers collective	Farmers	Farmers	No change
**Your Choice**			

*

Please choose the appropriate response for each item:

	○	○	○

{CECS1Area.question}

*

Only answer this question if the following conditions are met:
Answer was '' *or* '' at question ' [CECS5]' (Choice Set 5 Option A Option B None of the two Additional Payments compared to current levels +500 CHF per ha and year +1000 CHF per ha and year No change Collective with number of people you are in a group with 10 15 No change Monitoring in person or via digital tools In person Digital tools No change Discretion with the local government or farmers collective Farmers Farmers No change Your Choice $(document).on('ready pjax:scriptcomplete',function(){ // Identify this question var thisQuestion = $('#question{QID}'); // Move the radios $('.question-text table:eq(0) tr:last td:eq(2)', thisQuestion).append($('.subquestion-list .answers-list:eq(0) .answer-item:eq(0) *', thisQuestion)); $('.question-text table:eq(0) tr:last td:eq(4)', thisQuestion).append($('.subquestion-list .answers-list:eq(0) .answer-item:eq(1) *', thisQuestion)); $('.question-text table:eq(0) tr:last td:eq(6)', thisQuestion).append($('.subquestion-list .answers-list:eq(0) .answer-item:eq(2) *', thisQuestion)); // Some classes for presentation $('.question-text table:eq(0) input:radio', thisQuestion).closest('td').addClass('answer-item radio-item text-center radio'); $('.question-text table:eq(0) .radio-item label', thisQuestion).show(); // Click event on the table cells $('.question-text table:eq(0) .radio-item', thisQuestion).on('click', function(e) { $('input:radio', this).trigger('click'); }); $('.question-text table:eq(0) input:radio', thisQuestion).on('click', function(e) { e.stopPropagation(); }); // Clean-up styles $('.answer-container', thisQuestion).hide(); $('.question-text table:eq(0) .label-text', thisQuestion).remove(); $('.question-text table:eq(0) .radio-text', thisQuestion).css({ 'cursor': 'pointer' }); }); ())

Please choose **only one** of the following:

- ◯ -10% or less
- ◯ 0 to -10%
- ◯ 0% (no change)
- ◯ 0 to +10%
- ◯ +10% or more

{CECS1Helpdesk.question}

Please choose **only one** of the following:

- ◯ Explanatory Video
- ◯ Explanatory Text

{CEDescriptionBasis.question}

{CEDescriptionOptions.question}

Only answer this question if the following conditions are met:
Answer was 'Explanatory Text' at question ' [CECS5Helpdesk]' ({CECS1Helpdesk.question} $(document).on('ready pjax:scriptcomplete',function(){ $('.button-item:contains("No answer") label').text('Hide Help'); });)

Choice Experiment Choice Set 6

Choice Set 6

	Option A	Option B	None of the two
**Additional Payments** compared to current levels	+1000 CHF per ha and year	+500 CHF per ha and year	No change
**Collective with** number of people you are in a group with	15	20	No change
**Monitoring** in person or via digital tools	In person	Digital tools	No change
**Discretion** with the local government or farmers collective	State	Farmers	No change
**Your Choice**			

*

Please choose the appropriate response for each item:

	○	○	○

{CECS1Area.question}

*

Only answer this question if the following conditions are met:
Answer was '' *or* '' at question ' [CECS6]' (Choice Set 6 Option A Option B None of the two Additional Payments compared to current levels +1000 CHF per ha and year +500 CHF per ha and year No change Collective with number of people you are in a group with 15 20 No change Monitoring in person or via digital tools In person Digital tools No change Discretion with the local government or farmers collective State Farmers No change Your Choice $(document).on('ready pjax:scriptcomplete',function(){ // Identify this question var thisQuestion = $('#question{QID}'); // Move the radios $('.question-text table:eq(0) tr:last td:eq(2)', thisQuestion).append($('.subquestion-list .answers-list:eq(0) .answer-item:eq(0) *', thisQuestion)); $('.question-text table:eq(0) tr:last td:eq(4)', thisQuestion).append($('.subquestion-list .answers-list:eq(0) .answer-item:eq(1) *', thisQuestion)); $('.question-text table:eq(0) tr:last td:eq(6)', thisQuestion).append($('.subquestion-list .answers-list:eq(0) .answer-item:eq(2) *', thisQuestion)); // Some classes for presentation $('.question-text table:eq(0) input:radio', thisQuestion).closest('td').addClass('answer-item radio-item text-center radio'); $('.question-text table:eq(0) .radio-item label', thisQuestion).show(); // Click event on the table cells $('.question-text table:eq(0) .radio-item', thisQuestion).on('click', function(e) { $('input:radio', this).trigger('click'); }); $('.question-text table:eq(0) input:radio', thisQuestion).on('click', function(e) { e.stopPropagation(); }); // Clean-up styles $('.answer-container', thisQuestion).hide(); $('.question-text table:eq(0) .label-text', thisQuestion).remove(); $('.question-text table:eq(0) .radio-text', thisQuestion).css({ 'cursor': 'pointer' }); }); ())

Please choose **only one** of the following:

- -10% or less
- 0 to -10%
- 0% (no change)
- 0 to +10%
- +10% or more

{CECS1Helpdesk.question}

Please choose **only one** of the following:

- Explanatory Video
- Explanatory Text

{CEDescriptionBasis.question}

{CEDescriptionOptions.question}

Only answer this question if the following conditions are met:
Answer was 'Explanatory Text' at question ' [CECS6Helpdesk]' ({CECS1Helpdesk.question} $(document).on('ready pjax:scriptcomplete',function(){ $('.button-item:contains("No answer") label').text('Hide Help'); });)

Choice Experiment - Choice Set 7

Choice Set 7

	**Option A**	**Option B**	**None of the two**
**Additional Payments** compared to current levels	+1500 CHF per ha and year	+500 CHF per ha and year	No change
**Collective with** number of people you are in a group with	10	3	No change
**Monitoring** in person or via digital tools	Digital tools	Digital tools	No change
**Discretion** with the local government or farmers collective	State	State	No change
**Your Choice**			

*

Please choose the appropriate response for each item:

	○	○	○

{CECS1Area.question}

*

Only answer this question if the following conditions are met:
Answer was '' *or* '' at question ' [CECS7]' (Choice Set 7 Option A Option B None of the two Additional Payments compared to current levels +1500 CHF per ha and year +500 CHF per ha and year No change Collective with number of people you are in a group with 10 3 No change Monitoring in person or via digital tools Digital tools Digital tools No change Discretion with the local government or farmers collective State State No change Your Choice $(document).on('ready pjax:scriptcomplete',function(){ // Identify this question var thisQuestion = $('#question{QID}'); // Move the radios $('.question-text table:eq(0) tr:last td:eq(2)', thisQuestion).append($('.subquestion-list .answers-list:eq(0) .answer-item:eq(0) *', thisQuestion)); $('.question-text table:eq(0) tr:last td:eq(4)', thisQuestion).append($('.subquestion-list .answers-list:eq(0) .answer-item:eq(1) *', thisQuestion)); $('.question-text table:eq(0) tr:last td:eq(6)', thisQuestion).append($('.subquestion-list .answers-list:eq(0) .answer-item:eq(2) *', thisQuestion)); // Some classes for presentation $('.question-text table:eq(0) input:radio', thisQuestion).closest('td').addClass('answer-item radio-item text-center radio'); $('.question-text table:eq(0) .radio-item label', thisQuestion).show(); // Click event on the table cells $('.question-text table:eq(0) .radio-item', thisQuestion).on('click', function(e) { $('input:radio', this).trigger('click'); }); $('.question-text table:eq(0) input:radio', thisQuestion).on('click', function(e) { e.stopPropagation(); }); // Clean-up styles $('.answer-container', thisQuestion).hide(); $('.question-text table:eq(0) .label-text', thisQuestion).remove(); $('.question-text table:eq(0) .radio-text', thisQuestion).css({ 'cursor': 'pointer' }); }); ())

Please choose **only one** of the following:

- ◯ -10% or less
- ◯ 0 to -10%
- ◯ 0% (no change)
- ◯ 0 to +10%
- ◯ +10% or more

{CECS1Helpdesk.question}

Please choose **only one** of the following:

- ◯ Explanatory Video
- ◯ Explanatory Text

{CEDescriptionBasis.question}

{CEDescriptionOptions.question}

Only answer this question if the following conditions are met:
Answer was 'Explanatory Text' at question ' [CECS7Helpdesk]' ({CECS1Helpdesk.question} $(document).on('ready pjax:scriptcomplete',function(){ $('.button-item:contains("No answer") label').text('Hide Help'); });)

Choice Experiment - Choice Set 8

Choice Set 8

	Option A	Option B	None of the two
**Additional Payments** compared to current levels	+1500 CHF per ha and year	+1000 CHF per ha and year	No change
**Collective with** number of people you are in a group with	15	20	No change
**Monitoring** in person or via digital tools	In person	In person	No change
**Discretion** with the local government or farmers collective	Farmers	State	No change
**Your Choice**			

*

Please choose the appropriate response for each item:

	◯	◯	◯

{CECS1Area.question}

*

Only answer this question if the following conditions are met:
Answer was '' *or* '' at question ' [CECS8]' (Choice Set 8 Option A Option B None of the two Additional Payments compared to current levels +1500 CHF per ha and year +1000 CHF per ha and year No change Collective with number of people you are in a group with 15 20 No change Monitoring in person or via digital tools In person In person No change Discretion with the local government or farmers collective Farmers State No change Your Choice $(document).on('ready pjax:scriptcomplete',function(){ // Identify this question var thisQuestion = $('#question{QID}'); // Move the radios $('.question-text table:eq(0) tr:last td:eq(2)', thisQuestion).append($('.subquestion-list .answers-list:eq(0) .answer-item:eq(0) *', thisQuestion)); $('.question-text table:eq(0) tr:last td:eq(4)', thisQuestion).append($('.subquestion-list .answers-list:eq(0) .answer-item:eq(1) *', thisQuestion)); $('.question-text table:eq(0) tr:last td:eq(6)', thisQuestion).append($('.subquestion-list .answers-list:eq(0) .answer-item:eq(2) *', thisQuestion)); // Some classes for presentation $('.question-text table:eq(0) input:radio', thisQuestion).closest('td').addClass('answer-item radio-item text-center radio'); $('.question-text table:eq(0) .radio-item label', thisQuestion).show(); // Click event on the table cells $('.question-text table:eq(0) .radio-item', thisQuestion).on('click', function(e) { $('input:radio', this).trigger('click'); }); $('.question-text table:eq(0) input:radio', thisQuestion).on('click', function(e) { e.stopPropagation(); }); // Clean-up styles $('.answer-container', thisQuestion).hide(); $('.question-text table:eq(0) .label-text', thisQuestion).remove(); $('.question-text table:eq(0) .radio-text', thisQuestion).css({ 'cursor': 'pointer' }); }); ())

Please choose **only one** of the following:

- ◯ -10% or less
- ◯ 0 to -10%
- ◯ 0% (no change)
- ◯ 0 to +10%
- ◯ +10% or more

{CECS1Helpdesk.question}

Please choose **only one** of the following:

- ◯ Explanatory Video
- ◯ Explanatory Text

{CEDescriptionBasis.question}

{CEDescriptionOptions.question}

Only answer this question if the following conditions are met:
Answer was 'Explanatory Text' at question ' [CECS8Helpdesk]' ({CECS1Helpdesk.question} $(document).on('ready pjax:scriptcomplete',function(){ $('.button-item:contains("No answer") label').text('Hide Help'); });)

Choice Experiment - Choice Set 9

Choice Set 9

	Option A	Option B	None of the two
**Additional Payments** compared to current levels	+1500 CHF per ha and year	+500 CHF per ha and year	No change
**Collective with** number of people you are in a group with	10	3	No change
**Monitoring** in person or via digital tools	Digital tools	Digital tools	No change
**Discretion** with the local government or farmers collective	State	State	No change
**Your Choice**			

*

Please choose the appropriate response for each item:

	◯	◯	◯

{CECS1Area.question}

*

Only answer this question if the following conditions are met:
Answer was '' *or* '' at question ' [CECS9]' (Choice Set 9 Option A Option B None of the two Additional Payments compared to current levels +1500 CHF per ha and year +500 CHF per ha and year No change Collective with number of people you are in a group with 10 3 No change Monitoring in person or via digital tools Digital tools Digital tools No change Discretion with the local government or farmers collective State State No change Your Choice $(document).on('ready pjax:scriptcomplete',function(){ // Identify this question var thisQuestion = $('#question{QID}'); // Move the radios $('.question-text table:eq(0) tr:last td:eq(2)', thisQuestion).append($('.subquestion-list .answers-list:eq(0) .answer-item:eq(0) *', thisQuestion)); $('.question-text table:eq(0) tr:last td:eq(4)', thisQuestion).append($('.subquestion-list .answers-list:eq(0) .answer-item:eq(1) *', thisQuestion)); $('.question-text table:eq(0) tr:last td:eq(6)', thisQuestion).append($('.subquestion-list .answers-list:eq(0) .answer-item:eq(2) *', thisQuestion)); // Some classes for presentation $('.question-text table:eq(0) input:radio', thisQuestion).closest('td').addClass('answer-item radio-item text-center radio'); $('.question-text table:eq(0) .radio-item label', thisQuestion).show(); // Click event on the table cells $('.question-text table:eq(0) .radio-item', thisQuestion).on('click', function(e) { $('input:radio', this).trigger('click'); }); $('.question-text table:eq(0) input:radio', thisQuestion).on('click', function(e) { e.stopPropagation(); }); // Clean-up styles $('.answer-container', thisQuestion).hide(); $('.question-text table:eq(0) .label-text', thisQuestion).remove(); $('.question-text table:eq(0) .radio-text', thisQuestion).css({ 'cursor': 'pointer' }); }); ())

Please choose **only one** of the following:

- ◯ -10% or less
- ◯ 0 to -10%
- ◯ 0% (no change)
- ◯ 0 to +10%
- ◯ +10% or more

{CECS1Helpdesk.question}

Please choose **only one** of the following:

- ◯ Explanatory Video
- ◯ Explanatory Text

{CEDescriptionBasis.question}

{CEDescriptionOptions.question}

Only answer this question if the following conditions are met:
Answer was 'Explanatory Text' at question ' [CECS9Helpdesk]' ({CECS1Helpdesk.question} $(document).on('ready pjax:scriptcomplete',function(){ $('.button-item:contains("No answer") label').text('Hide Help'); });)

Choice Experiment - Choice Set 10

Choice Set 10

	**Option A**	**Option B**	**None of the two**
**Additional Payments** compared to current levels	+1500 CHF per ha and year	+500 CHF per ha and year	No change
**Collective with** number of people you are in a group with	10	3	No change
**Monitoring** in person or via digital tools	Digital tools	Digital tools	No change
**Discretion** with the local government or farmers collective	State	State	No change
Your Choice			

*

Please choose the appropriate response for each item:

	◯	◯	◯

{CECS1Area.question}

*

Only answer this question if the following conditions are met:
Answer was '' *or* '' at question ' [CECS10]' (Choice Set 10 Option A Option B None of the two Additional Payments compared to current levels +1500 CHF per ha and year +500 CHF per ha and year No change Collective with number of people you are in a group with 10 3 No change Monitoring in person or via digital tools Digital tools Digital tools No change Discretion with the local government or farmers collective State State No change Your Choice $(document).on('ready pjax:scriptcomplete',function(){ // Identify this question var thisQuestion = $('#question{QID}'); // Move the radios $('.question-text table:eq(0) tr:last td:eq(2)', thisQuestion).append($('.subquestion-list .answers-list:eq(0) .answer-item:eq(0) *', thisQuestion)); $('.question-text table:eq(0) tr:last td:eq(4)', thisQuestion).append($('.subquestion-list .answers-list:eq(0) .answer-item:eq(1) *', thisQuestion)); $('.question-text table:eq(0) tr:last td:eq(6)', thisQuestion).append($('.subquestion-list .answers-list:eq(0) .answer-item:eq(2) *', thisQuestion)); // Some classes for presentation $('.question-text table:eq(0) input:radio', thisQuestion).closest('td').addClass('answer-item radio-item text-center radio'); $('.question-text table:eq(0) .radio-item label', thisQuestion).show(); // Click event on the table cells $('.question-text table:eq(0) .radio-item', thisQuestion).on('click', function(e) { $('input:radio', this).trigger('click'); }); $('.question-text table:eq(0) input:radio', thisQuestion).on('click', function(e) { e.stopPropagation(); }); // Clean-up styles $('.answer-container', thisQuestion).hide(); $('.question-text table:eq(0) .label-text', thisQuestion).remove(); $('.question-text table:eq(0) .radio-text', thisQuestion).css({ 'cursor': 'pointer' }); }); ())

Please choose **only one** of the following:

- ◯ -10% or less
- ◯ 0 to -10%
- ◯ 0% (no change)
- ◯ 0 to +10%
- ◯ +10% or more

{CECS1Helpdesk.question}

Please choose **only one** of the following:

- ◯ Explanatory Video
- ◯ Explanatory Text

{CEDescriptionBasis.question}

{CEDescriptionOptions.question}

Only answer this question if the following conditions are met:
Answer was 'Explanatory Text' at question ' [CECS10Helpdesk]' ({CECS1Helpdesk.question} $(document).on('ready pjax:scriptcomplete',function(){ $('.button-item:contains("No answer") label').text('Hide Help'); });)

Choice Experiment Debriefing questions

By filling out the choice cards you have revealed to us which combination of chraracteristics of the agri-environmental scheme you prefer. Under the "best" conditions (meaning the characteristics you preferred most) which of the following things do you think apply?

Under such a scheme...

*

Please choose the appropriate response for each item:

	Does not apply at all	Does not apply	Neutral	Applies	Applies very much
**There is an additional payment for my additional efforts**					
**There is an additional payment without much more effort**					
**There is increased production**					
**There is decreased production**					
**I can learn from and have exchanges with other farmers**					
**I lose part of my agency and control over my management to the group**					
**I can show my skills as a farmer**					
**Biodiversity levels increase**					
**My production system becomes more resilient (e.g. to droughts)**					
**We can diversify our production or mobilize other resources (apart from the biodiversity conservation) as a group**					
**We have more weight in negotiations as a group**					
**I spend much time and energy on the scheme**					
**Other**					

If you chose "Other", please specify: *

Only answer this question if the following conditions are met:
Answer was 'Neutral' *or* 'Applies' *or* 'Applies very much' at question ' [CEAnticipatedOutcome]' (By filling out the choice cards you have revealed to us which combination of chraracteristics of the agri-environmental scheme you prefer. Under the "best" conditions (meaning the characteristics you preferred most) which of the following things do you think apply? Under such a scheme... (Other))

Please write your answer here:

Can you explain why you chose "none of the two" (C) for all of the questions? *

Only answer this question if the following conditions are met:
Answer was '' at question ' [CECS1]' (Choice Set 1 Option A Option B None of the two Additional Payments compared to current levels +1500 CHF per ha and year +500 CHF per ha and year No change Collective with number of people you are in a group with 10 3 No change Monitoring in person or via digital tools Digital tools Digital tools No change Discretion with the local government or farmers collective State State No change Your Choice $(document).on('ready pjax:scriptcomplete',function(){ // Identify this question var thisQuestion = $('#question{QID}'); // Move the radios $('.question-text table:eq(0) tr:last td:eq(2)', thisQuestion).append($('.subquestion-list .answers-list:eq(0) .answer-item:eq(0) *', thisQuestion)); $('.question-text table:eq(0) tr:last td:eq(4)', thisQuestion).append($('.subquestion-list .answers-list:eq(0) .answer-item:eq(1) *', thisQuestion)); $('.question-text table:eq(0) tr:last td:eq(6)', thisQuestion).append($('.subquestion-list .answers-list:eq(0) .answer-item:eq(2) *', thisQuestion)); // Some classes for presentation $('.question-text table:eq(0) input:radio', thisQuestion).closest('td').addClass('answer-item radio-item text-center radio'); $('.question-text table:eq(0) .radio-item label', thisQuestion).show(); // Click event on the table cells $('.question-text table:eq(0) .radio-item', thisQuestion).on('click', function(e) { $('input:radio', this).trigger('click'); }); $('.question-text table:eq(0) input:radio', thisQuestion).on('click', function(e) { e.stopPropagation(); }); // Clean-up styles $('.answer-container', thisQuestion).hide(); $('.question-text table:eq(0) .label-text', thisQuestion).remove(); $('.question-text table:eq(0) .radio-text', thisQuestion).css({ 'cursor': 'pointer' }); }); ())
and Answer was '' at question ' [CECS2]' (Choice Set 2 Option A Option B None of the two Additional Payments compared to current levels +1500 CHF per ha and year +500 CHF per ha and year No change Collective with number of people you are in a group with 10 3 No change Monitoring in person or via digital tools Digital tools Digital tools No change Discretion with the local government or farmers collective State State No change Your Choice $(document).on('ready pjax:scriptcomplete',function(){ // Identify this question var thisQuestion = $('#question{QID}'); // Move the radios $('.question-text table:eq(0) tr:last td:eq(2)', thisQuestion).append($('.subquestion-list .answers-list:eq(0) .answer-item:eq(0) *', thisQuestion)); $('.question-text table:eq(0) tr:last td:eq(4)', thisQuestion).append($('.subquestion-list .answers-list:eq(0) .answer-item:eq(1) *', thisQuestion)); $('.question-text table:eq(0) tr:last td:eq(6)', thisQuestion).append($('.subquestion-list .answers-list:eq(0) .answer-item:eq(2) *', thisQuestion)); // Some classes for presentation $('.question-text table:eq(0) input:radio', thisQuestion).closest('td').addClass('answer-item radio-item text-center radio'); $('.question-text table:eq(0) .radio-item label', thisQuestion).show(); // Click event on the table cells $('.question-text table:eq(0) .radio-item', thisQuestion).on('click', function(e) { $('input:radio', this).trigger('click'); }); $('.question-text table:eq(0) input:radio', thisQuestion).on('click', function(e) { e.stopPropagation(); }); // Clean-up styles $('.answer-container', thisQuestion).hide(); $('.question-text table:eq(0) .label-text', thisQuestion).remove(); $('.question-text table:eq(0) .radio-text', thisQuestion).css({ 'cursor': 'pointer' }); }); ())
and Answer was '' at question ' [CECS3]' (Choice Set 3 Option A Option B None of the two Additional Payments compared to current levels +1000 CHF per ha and year +500 CHF per ha and year No change Collective with number of people you are in a group with 1 3 No change Monitoring in person or via digital tools In person In person No change Discretion with the local government or farmers collective Farmers Farmers No change Your Choice $(document).on('ready pjax:scriptcomplete',function(){ // Identify this question var thisQuestion = $('#question{QID}'); // Move the radios $('.question-text table:eq(0) tr:last td:eq(2)', thisQuestion).append($('.subquestion-list .answers-list:eq(0) .answer-item:eq(0) *', thisQuestion)); $('.question-text table:eq(0) tr:last td:eq(4)', thisQuestion).append($('.subquestion-list .answers-list:eq(0) .answer-item:eq(1) *', thisQuestion)); $('.question-text table:eq(0) tr:last td:eq(6)', thisQuestion).append($('.subquestion-list .answers-list:eq(0) .answer-item:eq(2) *', thisQuestion)); // Some classes for presentation $('.question-text table:eq(0) input:radio', thisQuestion).closest('td').addClass('answer-item radio-item text-center radio'); $('.question-text table:eq(0) .radio-item label', thisQuestion).show(); // Click event on the table cells $('.question-text table:eq(0) .radio-item', thisQuestion).on('click', function(e) { $('input:radio', this).trigger('click'); }); $('.question-text table:eq(0) input:radio', thisQuestion).on('click', function(e) { e.stopPropagation(); }); // Clean-up styles $('.answer-container', thisQuestion).hide(); $('.question-text table:eq(0) .label-text', thisQuestion).remove(); $('.question-text table:eq(0) .radio-text', thisQuestion).css({ 'cursor': 'pointer' }); }); ())
and Answer was '' at question ' [CECS4]' (Choice Set 4 Option A Option B None of the two Additional Payments compared to current levels +500 CHF per ha and year +1000 CHF per ha and year No change Collective with number of people you are in a group with 6 3 No change Monitoring In person or via digital tools In person In person No change Discretion With the local government or farmers collective State State No change Your Choice $(document).on('ready pjax:scriptcomplete',function(){ // Identify this question var thisQuestion = $('#question{QID}'); // Move the radios $('.question-text table:eq(0) tr:last td:eq(2)', thisQuestion).append($('.subquestion-list .answers-list:eq(0) .answer-item:eq(0) *', thisQuestion)); $('.question-text table:eq(0) tr:last td:eq(4)', thisQuestion).append($('.subquestion-list .answers-list:eq(0) .answer-item:eq(1) *', thisQuestion)); $('.question-text table:eq(0) tr:last td:eq(6)', thisQuestion).append($('.subquestion-list .answers-list:eq(0) .answer-item:eq(2) *', thisQuestion)); // Some classes for presentation $('.question-text table:eq(0) input:radio', thisQuestion).closest('td').addClass('answer-item radio-item text-center radio'); $('.question-text table:eq(0) .radio-item label', thisQuestion).show(); // Click event on the table cells $('.question-text table:eq(0) .radio-item', thisQuestion).on('click', function(e) { $('input:radio', this).trigger('click'); }); $('.question-text table:eq(0) input:radio', thisQuestion).on('click', function(e) { e.stopPropagation(); }); // Clean-up styles $('.answer-container', thisQuestion).hide(); $('.question-text table:eq(0) .label-text', thisQuestion).remove(); $('.question-text table:eq(0) .radio-text', thisQuestion).css({ 'cursor': 'pointer' }); }); ())
and Answer was '' at question ' [CECS5]' (Choice Set 5 Option A Option B None of the two Additional Payments compared to current levels +500 CHF per ha and year +1000 CHF per ha and year No change Collective with number of people you are in a group with 10 15 No change Monitoring in person or via digital tools In person Digital tools No change Discretion with the local government or farmers collective Farmers Farmers No change Your Choice $(document).on('ready pjax:scriptcomplete',function(){ // Identify this question var thisQuestion = $('#question{QID}'); // Move the radios $('.question-text table:eq(0) tr:last td:eq(2)', thisQuestion).append($('.subquestion-list .answers-list:eq(0) .answer-item:eq(0) *', thisQuestion)); $('.question-text table:eq(0) tr:last td:eq(4)', thisQuestion).append($('.subquestion-list .answers-list:eq(0) .answer-item:eq(1) *',

thisQuestion)); $('.question-text table:eq(0) tr:last td:eq(6)', thisQuestion).append($('.subquestion-list .answers-list:eq(0) .answer-item:eq(2) *', thisQuestion)); // Some classes for presentation $('.question-text table:eq(0) input:radio', thisQuestion).closest('td').addClass('answer-item radio-item text-center radio'); $('.question-text table:eq(0) .radio-item label', thisQuestion).show(); // Click event on the table cells $('.question-text table:eq(0) .radio-item', thisQuestion).on('click', function(e) { $('input:radio', this).trigger('click'); }); $('.question-text table:eq(0) input:radio', thisQuestion).on('click', function(e) { e.stopPropagation(); }); // Clean-up styles $('.answer-container', thisQuestion).hide(); $('.question-text table:eq(0) .label-text', thisQuestion).remove(); $('.question-text table:eq(0) .radio-text', thisQuestion).css({ 'cursor': 'pointer' }); }); ()) *and* Answer was '' at question ' [CECS6]' (Choice Set 6 Option A Option B None of the two Additional Payments compared to current levels +1000 CHF per ha and year +500 CHF per ha and year No change Collective with number of people you are in a group with 15 20 No change Monitoring in person or via digital tools In person Digital tools No change Discretion with the local government or farmers collective State Farmers No change Your Choice $(document).on('ready pjax:scriptcomplete',function(){ // Identify this question var thisQuestion = $('#question{QID}'); // Move the radios $('.question-text table:eq(0) tr:last td:eq(2)', thisQuestion).append($('.subquestion-list .answers-list:eq(0) .answer-item:eq(0) *', thisQuestion)); $('.question-text table:eq(0) tr:last td:eq(4)', thisQuestion).append($('.subquestion-list .answers-list:eq(0) .answer-item:eq(1) *', thisQuestion)); $('.question-text table:eq(0) tr:last td:eq(6)', thisQuestion).append($('.subquestion-list .answers-list:eq(0) .answer-item:eq(2) *', thisQuestion)); // Some classes for presentation $('.question-text table:eq(0) input:radio', thisQuestion).closest('td').addClass('answer-item radio-item text-center radio'); $('.question-text table:eq(0) .radio-item label', thisQuestion).show(); // Click event on the table cells $('.question-text table:eq(0) .radio-item', thisQuestion).on('click', function(e) { $('input:radio', this).trigger('click'); }); $('.question-text table:eq(0) input:radio', thisQuestion).on('click', function(e) { e.stopPropagation(); }); // Clean-up styles $('.answer-container', thisQuestion).hide(); $('.question-text table:eq(0) .label-text', thisQuestion).remove(); $('.question-text table:eq(0) .radio-text', thisQuestion).css({ 'cursor': 'pointer' }); }); ()) *and* Answer was '' at question ' [CECS7]' (Choice Set 7 Option A Option B None of the two Additional Payments compared to current levels +1500 CHF per ha and year +500 CHF per ha and year No change Collective with number of people you are in a group with 10 3 No change Monitoring in person or via digital tools Digital tools Digital tools No change Discretion with the local government or farmers collective State State No change Your Choice $(document).on('ready pjax:scriptcomplete',function(){ // Identify this question var thisQuestion = $('#question{QID}'); // Move the radios $('.question-text table:eq(0) tr:last td:eq(2)', thisQuestion).append($('.subquestion-list .answers-list:eq(0) .answer-item:eq(0) *', thisQuestion)); $('.question-text table:eq(0) tr:last td:eq(4)', thisQuestion).append($('.subquestion-list .answers-list:eq(0) .answer-item:eq(1) *', thisQuestion)); $('.question-text table:eq(0) tr:last td:eq(6)', thisQuestion).append($('.subquestion-list .answers-list:eq(0) .answer-item:eq(2) *', thisQuestion)); // Some classes for presentation $('.question-text table:eq(0) input:radio', thisQuestion).closest('td').addClass('answer-item radio-item text-center radio'); $('.question-text table:eq(0) .radio-item label', thisQuestion).show(); // Click event on the table cells $('.question-text table:eq(0) .radio-item', thisQuestion).on('click', function(e) { $('input:radio', this).trigger('click'); }); $('.question-text table:eq(0) input:radio', thisQuestion).on('click', function(e) { e.stopPropagation(); }); // Clean-up styles $('.answer-container', thisQuestion).hide(); $('.question-text table:eq(0) .label-text', thisQuestion).remove(); $('.question-text table:eq(0) .radio-text', thisQuestion).css({ 'cursor': 'pointer' }); }); ()) *and* Answer was '' at question ' [CECS8]' (Choice Set 8 Option A Option B None of the two Additional Payments compared to current levels +1500 CHF per ha and year +1000 CHF per ha and year No change Collective with number of people you are in a group with 15 20 No change Monitoring in person or via digital tools In person In person No change Discretion with the local government or farmers collective Farmers State No change Your Choice $(document).on('ready pjax:scriptcomplete',function(){ // Identify this question var thisQuestion = $('#question{QID}'); // Move the radios $('.question-text table:eq(0) tr:last td:eq(2)', thisQuestion).append($('.subquestion-list .answers-list:eq(0) .answer-item:eq(0) *', thisQuestion)); $('.question-text table:eq(0) tr:last td:eq(4)', thisQuestion).append($('.subquestion-list .answers-list:eq(0) .answer-item:eq(1) *', thisQuestion)); $('.question-text table:eq(0) tr:last td:eq(6)', thisQuestion).append($('.subquestion-list .answers-list:eq(0) .answer-item:eq(2) *', thisQuestion)); // Some classes for presentation $('.question-text table:eq(0) input:radio', thisQuestion).closest('td').addClass('answer-item radio-item text-center radio'); $('.question-text table:eq(0) .radio-item label', thisQuestion).show(); // Click event on the table cells $('.question-text table:eq(0) .radio-item', thisQuestion).on('click', function(e) { $('input:radio', this).trigger('click'); }); $('.question-text table:eq(0) input:radio', thisQuestion).on('click', function(e) { e.stopPropagation(); }); // Clean-up styles $('.answer-container', thisQuestion).hide(); $('.question-text table:eq(0) .label-text', thisQuestion).remove(); $('.question-text table:eq(0) .radio-text', thisQuestion).css({ 'cursor': 'pointer' }); }); ()) *and* Answer was '' at question ' [CECS9]' (Choice Set 9 Option A Option B None of the two Additional Payments compared to current levels +1500 CHF per ha and year +500 CHF per ha and year No change Collective with number of people you are in a group with 10 3 No change Monitoring in person or via digital tools Digital tools Digital tools No change Discretion with the local government or farmers collective State State No change Your Choice $(document).on('ready pjax:scriptcomplete',function(){ // Identify this question var thisQuestion = $('#question{QID}'); // Move the radios $('.question-text table:eq(0) tr:last td:eq(2)', thisQuestion).append($('.subquestion-list .answers-list:eq(0) .answer-item:eq(0) *', thisQuestion)); $('.question-text table:eq(0) tr:last td:eq(4)', thisQuestion).append($('.subquestion-list .answers-list:eq(0) .answer-item:eq(1) *', thisQuestion)); $('.question-text table:eq(0) tr:last td:eq(6)', thisQuestion).append($('.subquestion-list .answers-list:eq(0) .answer-item:eq(2) *', thisQuestion)); // Some classes for presentation $('.question-text table:eq(0) input:radio', thisQuestion).closest('td').addClass('answer-item radio-item text-center radio'); $('.question-text table:eq(0) .radio-item label', thisQuestion).show(); // Click event on the table cells $('.question-text table:eq(0) .radio-item', thisQuestion).on('click', function(e) { $('input:radio', this).trigger('click'); }); $('.question-text table:eq(0) input:radio', thisQuestion).on('click', function(e) { e.stopPropagation(); }); // Clean-up styles $('.answer-container', thisQuestion).hide(); $('.question-text table:eq(0) .label-text', thisQuestion).remove(); $('.question-text table:eq(0) .radio-text', thisQuestion).css({ 'cursor': 'pointer' }); }); ()) *and* Answer was '' at question ' [CECS10]' (Choice Set 10 Option A Option B None of the two Additional Payments compared to current levels +1500 CHF per ha and year +500 CHF per ha and year No change Collective with number of people you are in a group with 10 3 No change Monitoring in person or via digital tools Digital tools Digital tools No change Discretion with the local government or farmers collective State State No change Your Choice $(document).on('ready pjax:scriptcomplete',function(){ // Identify this question var thisQuestion = $('#question{QID}'); // Move the radios $('.question-text table:eq(0) tr:last td:eq(2)', thisQuestion).append($('.subquestion-list .answers-list:eq(0) .answer-item:eq(0) *', thisQuestion)); $('.question-text table:eq(0) tr:last td:eq(4)', thisQuestion).append($('.subquestion-list .answers-list:eq(0)

.answer-item:eq(1) *', thisQuestion)); $('.question-text table:eq(0) tr:last td:eq(6)', thisQuestion).append($('.subquestion-list .answers-list:eq(0) .answer-item:eq(2) *', thisQuestion)); // Some classes for presentation $('.question-text table:eq(0) input:radio', thisQuestion).closest('td').addClass('answer-item radio-item text-center radio'); $('.question-text table:eq(0) .radio-item label', thisQuestion).show(); // Click event on the table cells $('.question-text table:eq(0) .radio-item', thisQuestion).on('click', function(e) { $('input:radio', this).trigger('click'); }); $('.question-text table:eq(0) input:radio', thisQuestion).on('click', function(e) { e.stopPropagation(); }); // Clean-up styles $('.answer-container', thisQuestion).hide(); $('.question-text table:eq(0) .label-text', thisQuestion).remove(); $('.question-text table:eq(0) .radio-text', thisQuestion).css({ 'cursor': 'pointer' }); }); ())

Select all that apply
Please choose **all** that apply:

- ☐ The additional payment is too low
- ☐ The goal of the scheme is not important to me
- ☐ The options were not realistic for my farm
- ☐ I do not want to be constrained in my farming practices, regardless of the compensation awarded
- ☐ No reason
- ☐ Other: ____________

Was the explanation of the agri-environmental scheme options (Additional payment, Collective with, Monitoring, Discretion) in the choice experiment **clear to you?** *

Please choose **only one** of the following:

- ○ Yes
- ○ No

Make a comment on your choice here:

Did you ignore any of the following elements when making decisions in the choice experiment? *

Please choose the appropriate response for each item:

	I did not ignore	I sometimes ignored	I always ignored
**Additional payments**	○	○	○
**Collective with**	○	○	○
**Monitoring**	○	○	○
**Discretion**	○	○	○

Cooperation and Attitudes on Cooperation

Please specify your cooperation activities with other farmers of the past, present, and potential future.

*

Please choose the appropriate response for each item:

	In the past	Ongoing	Planned or considered for the future	Never / not considered
**Environmental management (e.g. EXAMPLE)**	◯	◯	◯	◯
**Machinery sharing**	◯	◯	◯	◯
**Summering**	◯	◯	◯	◯
**Input purchases, Sales, Processing**	◯	◯	◯	◯
**Shared field work (e.g. for making silage)**	◯	◯	◯	◯
**Looking after someone’s farm so they can go on vacation**	◯	◯	◯	◯
**Exchanging knowledge, shared learning**	◯	◯	◯	◯
**Other**	◯	◯	◯	◯

If you have chosen "Other", please specify: *

Only answer this question if the following conditions are met:
Answer was 'In the past' *or* 'Ongoing' *or* 'Planned or considered for the future' at question ' [CooperationActivity]' (Please specify your cooperation activities with other farmers of the past, present, and potential future. (Other))

Please write your answer here:

Please state how much you agree with the following statements:

*

Please choose the appropriate response for each item:

	Strongly disagree	Disagree	Neutral	Agree	Strongly agree
I enjoy working with other farmers	◯	◯	◯	◯	◯
Achieving environmental goals in agriculture requires greater cooperation among farmers	◯	◯	◯	◯	◯
Cooperation with other farmers is generally difficult	◯	◯	◯	◯	◯
I frequently exchange ideas with other farmers on agricultural topics	◯	◯	◯	◯	◯

Environmental (Biodiversity) Attitudes

Please state how much you agree with the following statements:

*

Please choose the appropriate response for each item:

	Strongly disagree	Disagree	Neutral	Agree	Strongly agree
Biodiversity is declining worldwide	◯	◯	◯	◯	◯
Biodiversity is declining in my region	◯	◯	◯	◯	◯
Biodiversity is declining on my farm	◯	◯	◯	◯	◯
Biodiversity decline is an issue worldwide	◯	◯	◯	◯	◯
Biodiversity decline is an issue for my region	◯	◯	◯	◯	◯
Biodiversity decline is an issue for my production	◯	◯	◯	◯	◯
Global biodiversity is important to me	◯	◯	◯	◯	◯
Local/Regional biodiversity is important to me	◯	◯	◯	◯	◯
Biodiversity on my farm is important to me	◯	◯	◯	◯	◯

Is there a specific aspect of biodiversity that is important to you? *

Please choose **only one** of the following:

- Yes
- No

If you chose "yes", please specify: *

Only answer this question if the following conditions are met:
Answer was 'Yes' at question ' [BiodivAttitudeExtra]' (Is there a specific aspect of biodiversity that is important to you?)

Please write your answer here:

Perceptions

How would you rate the following statements regarding your role as a farmer in biodiversity conservation?

*

Please choose the appropriate response for each item:

	Strongly disagree	Disagree	Neutral	Agree	Strongly agree
**I can do something about biodiversity decline on my farm.**	◯	◯	◯	◯	◯
**When I encounter difficulties in my production, I can usually think of a solution**	◯	◯	◯	◯	◯
**My behavior as a farmer influences biodiversity.**	◯	◯	◯	◯	◯
**How successfully I can reduce biodiversity decline on the farm depends mainly on my skills as a farmer.**	◯	◯	◯	◯	◯
**I can solve production issues if I invest the necessary effort.**	◯	◯	◯	◯	◯
**I am confident that I can reduce biodiversity decline and at the same time produce successfully.**	◯	◯	◯	◯	◯
**Biodiversity decline is a problem I cannot change.**	◯	◯	◯	◯	◯

Preferences

How willing are you to take risks in the areas mentioned below?

*

Please choose the appropriate response for each item:

	0 = Not willing	1	2	3	4	5	6	7	8	9	10 = Very willing
**Production**	◯	◯	◯	◯	◯	◯	◯	◯	◯	◯	◯
**Market and prices**	◯	◯	◯	◯	◯	◯	◯	◯	◯	◯	◯
**Agriculture in general**	◯	◯	◯	◯	◯	◯	◯	◯	◯	◯	◯
**Collaboration**	◯	◯	◯	◯	◯	◯	◯	◯	◯	◯	◯

How willing are you to give up income that is beneficial for you/the farm today in order to benefit more from that in the future? *

Please choose the appropriate response for each item:

	0 = Not willing	1	2	3	4	5	6	7	8	9	10 = Very willing
**How willing are you to give up income that is beneficial for you/the farm today in order to benefit more from that in the future?**	○	○	○	○	○	○	○	○	○	○	○

Trust

How much do you agree that these people only have the best intentions? *

Please choose the appropriate response for each item:

	Strongly disagree	Disagree	Neutral	Agree	Strongly agree
**How much do you agree that people in general only have the best intentions?**	○	○	○	○	○
**How much do you agree that farmers in your region only have the best intentions?**	○	○	○	○	○
**How much do you agree that the regional government only has the best intentions?**	○	○	○	○	○

Social Network

Please state how much you agree with the following statements:

*

Please choose the appropriate response for each item:

	Strongly disagree	Disagree	Neutral	Agree	Strongly agree
**It is important to me what people around me think about the success of my farm and my farming skills**	○	○	○	○	○
**It is important to me to impress other farmers with my farm**	○	○	○	○	○
**I feel confirmed if I earn more than other farmers**	○	○	○	○	○
**On my farm, I want to produce more environmentally friendly than other farmers in my area**	○	○	○	○	○
**If other farmers in my environment earn more than I do, it bothers me**	○	○	○	○	○
**If other farmers in my environment implement biodiversity conservation measures, I want to implement such measures on my farm as well**	○	○	○	○	○

With how many people in the following groups did you discuss agricultural topics and biodiversity conservation measures within the last year? (Some people can appear in several categories)

*

Please choose the appropriate response for each item:

	None (0)	A few (1-5)	Some (5-10)	Many (10+)
**Other Farmers / people working in agriculture / association colleagues**	○	○	○	○
**Consultants/Extension service**	○	○	○	○
**Biodiversity Experts / Representatives of environmental organizations**	○	○	○	○
**Policy makers**	○	○	○	○
**Non-farmers in my community**	○	○	○	○
**Customers (direct marketing or retailers)**	○	○	○	○

How important are the opinions, attitudes and activities of these people when making decisions on your farm? (Some people can appear in several categories) *

Please choose the appropriate response for each item:

	Not at all important	Not important	Neutral	Important	Very important
**Other Farmers / people working in agriculture / association colleagues**	◯	◯	◯	◯	◯
**Consultants/Extension service**	◯	◯	◯	◯	◯
**Biodiversity Experts / Representatives of environmental organizations**	◯	◯	◯	◯	◯
**Policy makers**	◯	◯	◯	◯	◯
**Non-farmers in my community**	◯	◯	◯	◯	◯
**Customers (direct marketing or retailers)**	◯	◯	◯	◯	◯

Income Satisfaction and Production Orientation

How satisfied are you currently with your annual agricultural income (including direct payments, excluding off-farm income)?

*

Please choose **only one** of the following:

◯ Not at all satisfied
◯ Not satisfied
◯ Neutral
◯ Satisfied
◯ Very satisfied

How important is your agricultural income for you?

*

Please choose **only one** of the following:

◯ Not at all important
◯ Not important
◯ Neutral
◯ Important
◯ Very important

What is the share of agricultural income of total household income? *

Please choose **only one** of the following:

- ◯ 0-20 %
- ◯ 21-40%
- ◯ 41-60%
- ◯ 61-80%
- ◯ 81-100%

How important are the following criteria for your decision making? *

Please choose the appropriate response for each item:

	Not at all important	Not important	Neutral	Important	Very important
**Ensuring greatest crop yield**	◯	◯	◯	◯	◯
**Forestalling future problems**	◯	◯	◯	◯	◯
**Professional ambition to use best practices**	◯	◯	◯	◯	◯
**Contribution to a healthy environment**	◯	◯	◯	◯	◯
**Price of fodder**	◯	◯	◯	◯	◯
**Clean fields, (few weeds visible)**	◯	◯	◯	◯	◯
**Reduction of my workload**	◯	◯	◯	◯	◯
**Costs of inputs**	◯	◯	◯	◯	◯
**Subsidies for implementing sustainable methods**	◯	◯	◯	◯	◯
**Keeping a high biodiversity on my fields**	◯	◯	◯	◯	◯
**Achieving a high income (incl. direct payments)**	◯	◯	◯	◯	◯
**My image as a “good” farmer**	◯	◯	◯	◯	◯
**Consumers’ demand for products produced with a certain standard**	◯	◯	◯	◯	◯
**Long-term increase of soil fertility on my agricultural land**	◯	◯	◯	◯	◯
**Other farmers’ management methods**	◯	◯	◯	◯	◯
**Traditions**	◯	◯	◯	◯	◯

Outro

Thank you very much for your participation!

Your details and personal data will be kept strictly confidential and will be used exclusively for scientific purposes.

We will gladly send you a summary of the survey results if you have indicated your interest accordingly.

Do you have any final feedback or comments?

Please write your answer here:

Can we contact you again with a survey regarding your grassland yiel data? *

Please choose **only one** of the following:

- ◯ Yes
- ◯ No

We thank you very much for your participation!

For questions, please contact:

Viviane Fahrni,

Agricultural Economics and Policy Group (AECP), ETH Zurich

vfahrni@ethz.ch

You can now close the browser.

Best regards,

Viviane Fahrni (ETH Zurich)

Submit your survey.
Thank you for completing this survey.
