# Supplementary material for: Data on the stated willingness to accept collective agri-environmental schemes for biodiversity conservation of European grassland farmers
Source: Data Brief. 2026 Jun 17;67:112980. doi: 10.1016/j.dib.2026.112980 (PMC13315105; doi:10.1016/j.dib.2026.112980)
Supplement: Supplementary file 4 [file mmc4.pdf]

Landbruksdirektoratet
Eanandoallodirektoráhtta

Avtale om bruk av personopplysninger mellom Landbruksdirektoratet og GreeNet v/Ruralis – Institutt for rural og regionalforskning

Bakgrunn

Ruralis deltar i det internasjonale forskningsprosjektet «Grassland conservation across European landscapes protecting biodiversity and ecosystem services eith ecological networks» (GreeNet). Prosjektet ledes av Universität für Bodenkultur (BOKU) i Wien, hadde oppstart 1. april 2023 og vil pågå i tre år.

GreenNet skal utvikle scenarioer for bærekraftig bruk av europeiske grasarealer med utgangspunkt i differensiert bruk av arealer som skal lede til nye måter å utvikle og opprettholdes vernede landskap med slåtte- og beitemarker. Prosjektet har casestudier i Østerrike, Estland, Tyskland, Irland, Norge og Sveits. Casestudieområdet i Norge er Valdres.

Som del av prosjektet gjennomføres det en spørreundersøkelse blant bønder med grasproduksjon i samarbeidslandene, inkl. Norge. Utsendelsen av undersøkelsen er planlagt til januar/februar 2024. Undersøkelsen blir meldt og registrert hos Sikt, siden undersøkelsen vil inneholde personopplysninger.

Landbruksdirektoratet gir GreeNet v/Ruralis tilgang til epostadresser til søkere av produksjonstilskudd i jordbruket, som har søkt om tilskudd for kodene 210 – 212 i fagsystemet eStil PT. I den forbindelse er det vurdert at dette er personopplysninger, og det er derfor behov for å sikre at krav til vern av personopplysningene som det gis tilgang til, overholdes.

Forpliktelser for GreeNet v/Ruralis

Behandling av dataene GreeNet v/Ruralis mottar fra Landbruksdirektoratet for formål knyttet til forskningsprosjektet «Grassland conservation across European landscapes protecting biodiversity and ecosystem services eith ecological networks» (GreeNet), skal bare behandles for forskningsformål av GreeNet v/Ruralis og til det omtalte forskningsprosjektet. Dataene skal behandles i tråd med personopplysningsloven. Ved utlevering blir GreeNet behandlingsansvarlig av opplysningene i samsvar med personopplysningsloven og GDPR.

Ved publisering skal de omhandlede dataene anonymiseres.

For Landbruksdirektoratet	For GreeNet v/Ruralis
24.10.2023	24.10.2023
Linn Borsheim	Klaus Mittenzwei
