# Supplementary material for: Data on the stated willingness to accept collective agri-environmental schemes for biodiversity conservation of European grassland farmers
Source: Data Brief. 2026 Jun 17;67:112980. doi: 10.1016/j.dib.2026.112980 (PMC13315105; doi:10.1016/j.dib.2026.112980)
Supplement: Supplementary file 5 [file mmc5.pdf]

Sikt

Vurdering av behandling av personopplysninger

Referansenummer	Type vurdering	Dato
901807	Standard	18.01.2024

Tittel
GreeNet

Behandlingsansvarlig institusjon
Stiftelsen Ruralis institutt for rural- og regionalforskning

Prosjektansvarlig
Klaus Mittenzwei

Akademisk nivå
Forsknings-/dr.gradsprosjekt

Behandlingsperiode
01.01.2024 – 31.03.2026

Kategorier personopplysninger
Alminnelige

- Navn
- Kontaktinformasjon
- Nettidentifikator
- Bakgrunnsopplysninger, som i kombinasjon vil kunne identifisere en person

Lovlig grunnlag
Forskning i allmennhetens interesse, jf. GDPR art. 6(1)(e), jf. personopplysningsloven § 8

Konklusjon

> Vi har vurdert at behandlingen av personopplysninger i prosjektet er i samsvar med personvernregelverket, og kan starte. Opplysningene kan behandles til og med 31.03.2026.

Vi legger til grunn at behandlingen oppfyller kravene i personvernforordningen om riktighet art. 5.1(d), integritet og konfidensialitet art. 5.1.(f) og sikkerhet art.(32). Det betyr at du/dere må følge retningslinjene/lagringsguiden til behandlingsansvarlig institusjon for sikker behandling av forskningsdata.

OM VURDERINGEN Sikt har en avtale med institusjonen du forsker eller studerer ved. Denne avtalen innebærer at vi skal gi deg råd slik at behandlingen av personopplysninger i prosjektet ditt er lovlig etter personvernregelverket. Vi har nå vurdert at du har lovlig grunnlag til å behandle personopplysningene.

BEHANDLINGSGRUNNLAG - REKRUTTERING OG UTVALG Rekruttering Prosjektet vil behandle kontaktopplysninger og utvalgskriterier fordi det er nødvendig for forskning i allmennhetens interesse, jf. personvernforordningen art. 6 nr. 1 e), jf. personopplysningsloven § 8. Prosjektet gjør i den forbindelse nødvendige tiltak for å ivareta de registrertes rettigheter og friheter, jf. art. 89 nr. 1. I vår vurdering legger vi til grunn følgende beskrivelse av prosessen:

"Undersøkelsen vil bli gjennomført som en epost-undersøkelse, som vil bli utarbeidet og sendt ut med bruk av programvaren LimeSurvey. Uttrekket av respondenter baserer seg på et register som er tilrettelagt av Landbruksdirektoratet, og inneholder opplysninger om alle gårdbrukere i Norge. Dette innebærer blant annet informasjon om gårdens størrelse (areal) og antall dyr, samt kronebeløp på ulike mottatte tilskudd. Dette er opplysninger som offentlig tilgjengelig på data.norge.no, som vil bli koblet til spørreundersøkelsen. Disse variablene vil imidlertid anonymiseres slik at respondentene ikke vil bli direkte identifisert. Utover disse offentlige opplysningene har Landbruksdirektoratet godkjent utlevering av informasjon om gårdbrukernes epost-adresse (som skal brukes ved utsending). Registeret vil bli oversendt Ruralis, som videre vil gjøre et uttrekk på 3000 gårdbrukere blant de som har mottatt produksjonstilskudd, og som har søkt om tilskudd for kodene 210-212 i fagsystemet eStil PT. Informasjon om gårdbrukernes epost-adresse vil kun bli brukt i dette prosjektet, og epost-adressene vil bli slettet etter prosjektslutt."

Utvalget Den videre planlagte behandlingen av personopplysninger er nødvendig for å utføre en oppgave i allmennhetens interesse, jf. personvernforordningen art. 6 nr. 1 e).

Ifølge art. 6 nr. 3 b) skal grunnlaget for slik behandling fastsettes nærmere i nasjonal rett. Personopplysningsloven § 8 stadfester at behandling av personopplysninger for arkiv-, forsknings- eller statistikkformål er i allmennhetens interesse og kan gjøres på grunnlag av art. 6 nr. 1 e).

Prosjektet gjør nødvendige tiltak for å ivareta de registrertes rettigheter og friheter, jf. art. 89 nr. 1. I vår vurdering har vi lagt vekt på at forskningen har høy samfunnsnytte, som del av et større prosjekt, GreeNet, hvor formålet er å utvikle scenarioer for bærekraftig bruk av europeiske grasarealer med utgangspunkt i differensiert bruk av arealer som skal leder til nye måter å utvikle og opprettholde vernede landskaps med slått- og beitemarker. Videre skal opplysningene kun brukes til prosjektet, ikke andre formål Det skal bare samles inn opplysninger som er nødvendig for formålet Det skal kun registreres alminnelige personopplysninger Omfanget personopplysninger er relativt lite De registrerte får god informasjon om behandlingen og sine rettigheter Kun prosjektmedarbeidere har tilgang til opplysningene Personopplysninger minimeres fortløpende, og ingen vil kunne gjenkjennes i publikasjoner. Varigheten for behandling av personopplysninger er relativt kort

FØLG DIN INSTITUSJONS RETNINGSLINJER Det er institusjonen du er ansatt/student ved som avgjør hvordan du må lagre og sikre data i ditt prosjekt og hvilke databehandlere du kan bruke. Husk å bruke leverandører som din institusjon har avtale med (f.eks. ved skylagring, nettspørreskjema, videosamtale el.) .

Personverntjenester legger til grunn at behandlingen oppfyller kravene i personvernforordningen om riktighet (art. 5.1 d), integritet og konfidensialitet (art. 5.1. f) og sikkerhet (art. 32).

DATABEHANDLER Vi legger til grunn at behandlingen oppfyller kravene til bruk av databehandler, jf. personvernforordningen art. 28 og 29.

MELD VESENTLIGE ENDRINGER Dersom det skjer vesentlige endringer i behandlingen av personopplysninger, kan det være nødvendig å melde dette til oss ved å oppdatere meldeskjemaet. Se våre nettsider om hvilke endringer du må melde: https://sikt.no/melde-endringar-i-meldeskjema

OPPFØLGING AV PROSJEKTET Vi vil følge opp ved planlagt avslutning for å avklare om behandlingen av personopplysningene er avsluttet.

Lykke til med prosjektet!
