# Supplementary material for: Data on the stated willingness to accept collective agri-environmental schemes for biodiversity conservation of European grassland farmers
Source: Data Brief. 2026 Jun 17;67:112980. doi: 10.1016/j.dib.2026.112980 (PMC13315105; doi:10.1016/j.dib.2026.112980)
Supplement: Supplementary file 6 [file mmc6.pdf]

Leibniz-Zentrum für
Agrarlandschaftsforschung
(ZALF) e.V.

Leibniz-Zentrum für Agrarlandschaftsforschung (ZALF) e.V.
Eberswalder Straße 84, 15374 Müncheberg

Frau Dr. Sandra Utes
Arbeitsgruppe: Agrarökonomie und Ökosystemleistungen
Programmbereich 3 „Agrarlandschaftssysteme"
Leibniz Centre for Agricultural Landscape Research (ZALF)

Administrativer Direktor
Martin Jank
Administration und Services

T 0 33432 82 - 230
F 0 33432 82 - 215
E Martin.Jank@zalf.de

Eberswalder Straße 84
15374 Müncheberg
www.zalf.de

Müncheberg, 21.06.2024

Summary statement on the ethics application 1for the study " GreeNet Landwirte Umfrage"

Based on the description of the project provided, the designated members of the ZALF Ethics Committee gave the following feedback on the planned procedure.

The general assessment is that the project is regarded as uncritical in terms of ethical risks. The survey does not survey vulnerable groups and does not address sensitive content. Participation is voluntary and anonymous, there is no deception of the participants. Overall, from our point of view, there are no recognizable risks for the participants as well as the scientists conducting the project that go beyond the everyday level.

The following points which were given are positive and therefore encouraged:
- LimeSurvey on own server without saving participant Ips
- general consent given by participants
As a precautionary measure, we would like to point out that the DFG recommends that the data be stored for 10 years (see Guideline 17)

We ask for a short feedback on the following points:

- What exactly does this mean? "the data is anonymized further" (Kap. 3.2)?
- How high is the "incentivation" issued by lottery? Could this restrict the voluntary nature of participation?
- How long does it take to take part in the survey?
- Does the contacting of participants through the company “agri-direct” happen on a consent-basis?
- Does the consent to the survey include specific information about further use of answers in, for example, journal articles?
- How do you plan to feed back information without people giving their Email-addresses or contact information in the survey?
- Can you withdraw your consent to participate retrospectively? If so, in what form?
