# Supplementary material for: Data on the stated willingness to accept collective agri-environmental schemes for biodiversity conservation of European grassland farmers
Source: Data Brief. 2026 Jun 17;67:112980. doi: 10.1016/j.dib.2026.112980 (PMC13315105; doi:10.1016/j.dib.2026.112980)
Supplement: Supplementary file 7 [file mmc7.pdf]

Leibniz-Zentrum für Agrarlandschaftsforschung (ZALF) e.V.
Eberswalder Straße 84, 15374 Müncheberg

An den Administrativen Vorstand des ZALF

Dr.
Sandra Uthes
Programmbereich 3
AG LBW

T 0 33432 82 - 413
E uthes@zalf.de

Eberswalder Straße 84
15374 Müncheberg
www.zalf.de

Datum:
09.07.2024

Beantwortung der Hinweise von Ethikkomission (2024-06-21) und Datenschutzbeauftragtem (2024-07-08) im Zusammenhang mit der Landwirtebefragung im Projekt GreeNet

Sehr geehrter Herr Jank,

die von der Ethikkommission bzw. des Datenschutzbeauftragten des ZALF formulierten Hinweise möchte ich wie folgt beantworten:

- What exactly does this mean? "the data is anonymized further" (Kap. 3.2)?
This text was taken over from the Swiss ethical clearance application. The German survey is completely anonymous; therefore, no further anonymization takes place.

- How high is the "incentivation" issued by lottery? Could this restrict the voluntary nature of participation?
Following the recommendation of agri-direct, the incentivation was changed to a wish-voucher (Wunschgutschein) worth 15 Euro for each completed survey. According to agri-direct, without incentives, the required participation rate of 150 farmers cannot be achieved. Farmers dedicate their valuable time to answering the survey, and therefore deserve some form of compensation. Incentivation of such kind is standard practice in this field of research.

- How long does it take to take part in the survey?
20-30 min, this is mentioned on the entry page of the survey

- Does the contacting of participants through the company “agri-direct” happen on a consent-basis?
Yes

- Does the consent to the survey include specific information about further use of answers in, for example, journal articles?
We have added a corresponding text file to the questionnaire (“Information und Einwilligung”, Anlage 1).

- How do you plan to feedback information without people giving their Email-addresses or contact information in the survey?

This text passage was taken from the original Swiss survey, we have changed this question and respondents wishing to receive a feedback report can now fill in their email address.

- Can you withdraw your consent to participate retrospectively? If so, in what form?

Participation can only be withdrawn during the filling in of the survey. This is mentioned in the document "Information und Einwilligung" (Anlage 1).

****

1. Ethik: keine Einwände und Zustimmung zu dem zuvor Gesagten. Ergänzende Frage: Bis zu welchem Zeitpunkt kann die Einwilligung in die Teilnahme widerrufen werden (ich gehe davon aus, dass dies nur bis zum Abschicken der Antworten möglich sein wird)? Hierzu bedarf es auch einer transparenteren Information der Teilnehmer, über die Möglichkeit des Widerrufs bis zum Zeitpunkt x und die Möglichkeiten ab dem Zeitpunkt X.

   Es wird im neu hinzugefügten Dokument „Information und Einwilligung" darauf hingewiesen.

2. Datenschutz: in gebotener Kürze
   1. die übermittelte Einwilligung erfüllt nicht die Anforderungen an "Informiertheit" nach Art. 6 Abs. 1 a, 7 DS-GVO

      Die Informiertheit wird durch die Anlage 1 „Information und Einwilligung" gewährleistet.

   2. Ich kann keine DAtenschutzhinweise nach Art. 12, 13, 14 DS-GVO erkennen
      1. u.a. Nennung der "Datenquelle" unter vollständiger Firma (Unternehmensbezeichnung inkl. ladungsfähiger Adresse)

      Die Adresse des ZALF (wie in den Email Signaturen) ist im Dokument „Information und Einwilligung" eingefügt (Anlage 1).

   3. Wie wird der Nachweis geführt, dass agri-direct die Daten rechtmäßig erhoben und zur Verfügung gestellt hat (siehe Marcus)

      Agri-direct verwendet folgenden Disclaimer in der Email-Einladung zum Fragebogen:

Die AgriDirect Deutschland GmbH ist Versender dieses Newsletters. Die Inhalte sind von Dritten erstellt. AgriDirect übernimmt für den Inhalt keine Verantwortung oder Haftung. Die Umfrage wurde vom Leibniz-Zentrum für Agrarlandschaftsforschung (ZALF) e.V. erstellt und ist alleine für den Inhalt verantwortlich. Ihre Daten sind lediglich beim Versender gespeichert, ihre Antworten werden anonym übermittelt. Sie können der Nutzung Ihrer Adresse zu Werbezwecken jederzeit schriftlich widersprechen. Verantwortlicher lt. Art. 4 DSGVO: AgriDirect Deutschland GmbH, Dülkener Str. 56, 41747 Viersen, info@agridirect.de.

4. Wer von den wissenschaftl. "Partnern" erhält Zugang zu welchen Daten?

   Das ZALF und ETH Zürich werden zusammen die anonymen Daten für eine Publikation auswerten.

5. Wie wird "Anonymität" im Einzelfall sichergestellt, bei Kombination aus "Betriebsmerkmalen" und persönlichen Angaben, etwa in PLZ-Gebieten mit wenigen (einem) Landwirtschaftsbetrieb(en)?

   Wir haben die Frage geändert, es wird jetzt nach dem Bundesland gefragt.

6. Wie soll die Zusendung des Summary Reports unter Beibehaltung der Anonymität erfolgen?

   Die Frage wurde geändert; die Probanden werden jetzt gebeten, bei dieser Frage ihre Email anzugeben, wenn sie den Bericht zugeschickt bekommen möchten.

7. Welche Daten werden gelöscht, um vollständige Anonymität zu gewährleisten?

   Die Befragung ist anonym. Es werden keine Daten gelöscht.

8. Zu bedenken ist, dass ggf. eine "Gemeinsame Verantwortlichkeit" nach Art. 26 DS-GVO zwischen agri-direct und mind. dem ZALF vorliegen kann, auch wenn das ZALF zu keinem Zeitpunkt Zugang zu personenbezogenen Daten erhalten sollte. Hier kann eine Vorfilterung durch Definition von Ein- und Ausschlusskriterien für die Teilnahme als Bestimmung von "Zweck und Mittel" der Verarbeitung ausreichend sein (EuGH Zeugen Jehovas und Wirtschaftsakademie Schleswig-Holstein)

   Siehe Email-disclaimer (Punkt 3)

Mit freundlichen Grüßen

Dr. Sandra Uthes

Anlagen
Anlage 1: Information und Einwilligung
